# Haplotype-resolved nonaploid genome provides insights into *in vitro* flowering in bamboos

**DOI:** 10.1093/hr/uhae250

**Published:** 2024-09-04

**Authors:** Yu-Jiao Wang, Cen Guo, Lei Zhao, Ling Mao, Xiang-Zhou Hu, Yi-Zhou Yang, Ke-Cheng Qian, Peng-Fei Ma, Zhen-Hua Guo, De-Zhu Li

**Affiliations:** Germplasm Bank of Wild Species & Yunnan Key Laboratory of Crop Wild Relatives Omics, Kunming Institute of Botany, Chinese Academy of Sciences, 132 Lanhei Road, Panlong District, Kunming, Yunnan 650201, China; State Key Laboratory of Plant Diversity and Specialty Crops, Kunming Institute of Botany, Chinese Academy of Sciences, 132 Lanhei Road, Panlong District, Kunming, Yunnan 650201, China; Germplasm Bank of Wild Species & Yunnan Key Laboratory of Crop Wild Relatives Omics, Kunming Institute of Botany, Chinese Academy of Sciences, 132 Lanhei Road, Panlong District, Kunming, Yunnan 650201, China; Center for Integrative Conservation & Yunnan Key Laboratory for the Conservation of Tropical Rainforests and Asian Elephants, Xishuangbanna Tropical Botanical Garden, Chinese Academy of Sciences, Menglun, Yunnan 666303, China; Germplasm Bank of Wild Species & Yunnan Key Laboratory of Crop Wild Relatives Omics, Kunming Institute of Botany, Chinese Academy of Sciences, 132 Lanhei Road, Panlong District, Kunming, Yunnan 650201, China; State Key Laboratory of Plant Diversity and Specialty Crops, Kunming Institute of Botany, Chinese Academy of Sciences, 132 Lanhei Road, Panlong District, Kunming, Yunnan 650201, China; Germplasm Bank of Wild Species & Yunnan Key Laboratory of Crop Wild Relatives Omics, Kunming Institute of Botany, Chinese Academy of Sciences, 132 Lanhei Road, Panlong District, Kunming, Yunnan 650201, China; Center for Integrative Conservation & Yunnan Key Laboratory for the Conservation of Tropical Rainforests and Asian Elephants, Xishuangbanna Tropical Botanical Garden, Chinese Academy of Sciences, Menglun, Yunnan 666303, China; Kunming College of Life Science, University of Chinese Academy of Sciences,132 Lanhei Road, Panlong District, Kunming, Yunnan 650201, China; Germplasm Bank of Wild Species & Yunnan Key Laboratory of Crop Wild Relatives Omics, Kunming Institute of Botany, Chinese Academy of Sciences, 132 Lanhei Road, Panlong District, Kunming, Yunnan 650201, China; Center for Integrative Conservation & Yunnan Key Laboratory for the Conservation of Tropical Rainforests and Asian Elephants, Xishuangbanna Tropical Botanical Garden, Chinese Academy of Sciences, Menglun, Yunnan 666303, China; Kunming College of Life Science, University of Chinese Academy of Sciences,132 Lanhei Road, Panlong District, Kunming, Yunnan 650201, China; Germplasm Bank of Wild Species & Yunnan Key Laboratory of Crop Wild Relatives Omics, Kunming Institute of Botany, Chinese Academy of Sciences, 132 Lanhei Road, Panlong District, Kunming, Yunnan 650201, China; Center for Integrative Conservation & Yunnan Key Laboratory for the Conservation of Tropical Rainforests and Asian Elephants, Xishuangbanna Tropical Botanical Garden, Chinese Academy of Sciences, Menglun, Yunnan 666303, China; Kunming College of Life Science, University of Chinese Academy of Sciences,132 Lanhei Road, Panlong District, Kunming, Yunnan 650201, China; Germplasm Bank of Wild Species & Yunnan Key Laboratory of Crop Wild Relatives Omics, Kunming Institute of Botany, Chinese Academy of Sciences, 132 Lanhei Road, Panlong District, Kunming, Yunnan 650201, China; Kunming College of Life Science, University of Chinese Academy of Sciences,132 Lanhei Road, Panlong District, Kunming, Yunnan 650201, China; Germplasm Bank of Wild Species & Yunnan Key Laboratory of Crop Wild Relatives Omics, Kunming Institute of Botany, Chinese Academy of Sciences, 132 Lanhei Road, Panlong District, Kunming, Yunnan 650201, China; State Key Laboratory of Plant Diversity and Specialty Crops, Kunming Institute of Botany, Chinese Academy of Sciences, 132 Lanhei Road, Panlong District, Kunming, Yunnan 650201, China; Germplasm Bank of Wild Species & Yunnan Key Laboratory of Crop Wild Relatives Omics, Kunming Institute of Botany, Chinese Academy of Sciences, 132 Lanhei Road, Panlong District, Kunming, Yunnan 650201, China; State Key Laboratory of Plant Diversity and Specialty Crops, Kunming Institute of Botany, Chinese Academy of Sciences, 132 Lanhei Road, Panlong District, Kunming, Yunnan 650201, China; Germplasm Bank of Wild Species & Yunnan Key Laboratory of Crop Wild Relatives Omics, Kunming Institute of Botany, Chinese Academy of Sciences, 132 Lanhei Road, Panlong District, Kunming, Yunnan 650201, China

## Abstract

Woody bamboos (Bambusoideae) are renowned for its polyploidy and rare flowering. *Bambusa odashimae* is one of the bamboo species with the highest chromosome count (104) in the subfamily and has the highest heterozygosity of all sequenced bamboo genomes so far. Compared with other bamboo species, it can efficiently utilize exogenous hormones to regulate *in vitro* flowering, providing valuable insights into the hormonal regulation of bamboo flowering. Here, we generated the haplotype-resolved genome assembly of *B. odashimae*, despite the complexity and high chromosome number, supplemented by thirty-three transcriptomes from eleven developmental periods using a tissue culture system. The assembled genome can be divided into Haplotype I, Haplotype II, and Haplotype III, each containing A, B, and C subgenomes. Haplotype I may be derived from *Dendrocalamus* whereas Haplotypes II and III are closely related to *Bambusa*, indicating that *B. odashimae* has an origin involving both intergeneric and interspecific hybridizations. The high heterozygosity renders the possibility to detect abundant allele-specific expression (ASE), with ASE genes enriched in cytokinin-related pathways, likely associated with efficient cytokinin-promoted flowering. Notably, we found that the *CONSTAN*S (*CO*) genes were potentially key regulators of *in vitro* flowering in *B. odashimae*. Overall, based on the *in vitro* system combined with a high-quality reference genome, our study provides critical insights into the origin of this nonaploid bamboo and links hybridization and *in vitro* flowering in bamboos.

## Introduction

Bambusoideae (the bamboo subfamily) is a large clade of the grass family (Poaceae) and is widely distributed across East and Southeast Asia, Africa, and Latin America with some 1700 species [1]. As a fast-growing, renewable, perennial forest resource, woody bamboos have been increasingly used as a substitute for timber and has high potential for carbon fixation to mitigate global warming. However, unlike their herbaceous relatives, woody bamboo species are allopolyploid and have unique flowering behavior, including unpredictable flowering time, gregarious flowering after a long vegetative growth phase, and subsequent death of the bamboo clumps [2]. Understanding the polyploidization and flowering mechanisms of woody bamboos is of great theoretical and practical importance.

Polyploidy is a major driver of plant evolution. Bamboos represent a remarkable polyploid system, comprising an essentially diploid (2n = 2x = 20–24), herbaceous clade, two tetraploid (2n = 4x = 46–48), woody clades, and a hexaploid (2n = 6x = 70–72), paleotropical woody clade [3]. However, several species of the paleotropical woody bamboos (PWB), such as* B. odashimae* Hatus. ex Ohrnb. (syn. *B. edulis* (Odashima) Keng f.), *B. variostriata* (W. T. Lin) Chia & H. L. Fung, *B. gibboides* W. T. Lin, *B. prominens* H. L. Fung & S. Y. Sia, *B. xiashanensis* Chia & H. L. Fung, and *Cephalostachyum virgatum* (Munro) Kurz, have been suggested to possess 104 chromosomes in previous karyotype studies [4, 5]. This is the highest reported chromosome number in bamboos. The ploidal level of these bamboo species is not clear. Based on chromosome numbers of PWB [3, 6], it was inferred that they might exhibit nonaploidy (3n = 9x ≈ 104). Additionally, these species also showed aneuploidy. For example, *B. odashimae* has 96, 98, 102, or 104 chromosomes [4, 5]. Nevertheless, there is a lack of a reasonable explanation for the aneuploidy observed.

Polyploid plants have notable advantages, including stress resistance, increased vigor, etc. [7, 8]. *Bambusa odashimae* is highly popular due to its delicious bamboo shoots. Previous research has highlighted the robust viability of *B. odashimae* in culture medium and its exceptional capacity for flowering and rejuvenation [9, 10]. In bamboos, cytokinins promote flowering, whereas auxins facilitate rejuvenation during *in vitro* flowering [10, 11]. However, the molecular mechanisms of these hormones in regulating bamboo flowering remain unexplored. Furthermore, the absence of a genome sequence for this unique species has hindered detailed studies on *in vitro* flowering.

Early bamboo genome assemblies mainly relied on the Illumina platform, such as the genomes of *Phyllostachys edulis*, *Guadua angustifolia*, and *Raddia guianensis* [12, 13]. Recent advances in sequencing technology, single-molecule, real-time sequencing, have enabled to generate high-quality genomes. This technology has been used to assemble chromosome-level genomes for *Dendrocalamus latiflorus*, *Dendrocalamus sinicus,* and other bamboo species at different ploidy levels [3, 14–16]. Despite these advances, the genome of a nonaploid bamboo species has yet to be sequenced.

To investigate polyploidy and flowering patterns in *B. odashimae*, we reported a haplotype-resolved, chromosome-scale reference genome by using a combination of second-generation sequencing, Nanopore sequencing, and Hi-C technology. Through genome assembly and transcriptome sequencing at eleven stages in our unique *in vitro* system, we have gained valuable insights into the origin of a nonaploid bamboo and the hormonal regulation of *in vitro* flowering in woody bamboos.

## Results

### Genome assembly and annotation

We generated a total of 105 Gb of short paired-end reads. According to k-mer analysis, the estimated haploid genome size of *B. odashimae* was 1.04 Gb (Supplemental Fig. S1A), approximately one-third of the genome content estimated by flow cytometric analysis (3.38–3.64 Gb) (Table S1). And the estimated genome exhibits a heterozygosity rate of 7.3%.

**Figure 1:**
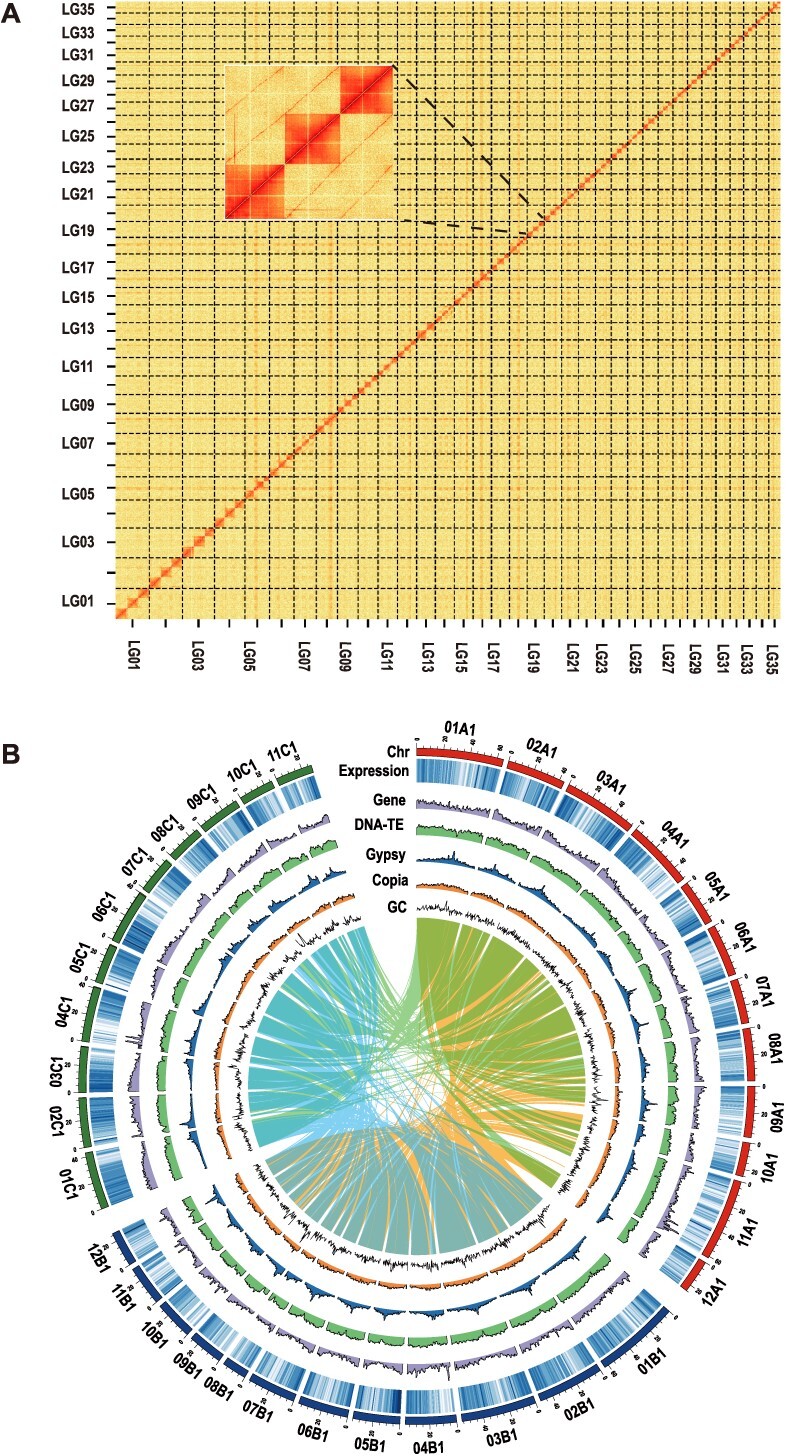
Genome assembly of *B. odashimae*. (A) Overview of the Hi-C heat map for assembled chromosomes. The intensity of pixels represents the count of Hi-C links between 300-kb windows on chromosomes. (B) The circular graph of the haplotype I genome (from the outer to the inner circle) represents the length (Mb) of chromosomes, gene expression characteristics, gene density, DNA-TE density, *Gypsy* density, *Copia* density, and GC content. All statistics were computed for windows of 100 kb. The syntenic blocks represent homoeologous connections among the subgenomes.

Through combining short paired-end reads, 176 Gb of ONT (Oxford Nanopore Technology) long reads, and 352 Gb of Hi-C paired-end reads, we generated a final merged chromosome-level assembly of 3.36 Gb with the contig N50 length of 14.81 Mb (Table S2). Using Hi-C data, 99.50% of the contigs were successfully categorized and ordered into 104 chromosome-scaffolds (Fig. 1A). These chromosomes vary in size from 16 to 61 Mb (Table S3). A total of 141 732 protein-coding genes were identified in the genome (Fig. 1B), 94.6% of which were functionally annotated (Table S4). Additionally, we identified 2.37 Gb of repetitive sequences, accounting for 70.54% of the genome. This includes 1898.41 Mb (55.18%) intact long terminal repeats (LTRs), with 563.85 Mb (16.39%) *Copia* and 618.31 Mb (17.97%) *Gypsy* elements (Table S5). To assess the completeness of the assembled *B. odashimae* genome, we performed Benchmarking Universal Single-Copy Orthologs (BUSCO) analysis and Core Eukaryotic Genes Mapping Approach (CEGMA). The results showed 98.39% BUSCO values (Table S6) and a 96.77% CEGMA completeness score (Table S7). The guanine-cytosine (GC) content of the *B. odashimae* genome was 44.5% (Supplemental Fig. S2A). Over 90% of the assembled transcripts could be mapped to the genome (Table S8), and the LTR Assembly Index (LAI) score is 16.6 (Supplemental Fig. S2B). Taken together, these results confirm the high quality of the assembly.

### Nonaploid origin

**Figure 2:**
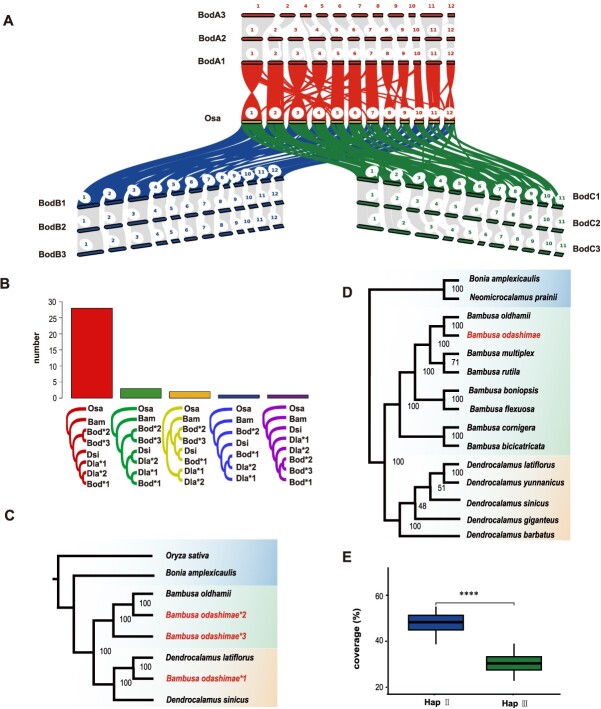
Haplome construction and phylogenetic tree. (A) Genomic synteny between *B. odashimae* and rice. The karyotype figure represents the syntenic relationship between *B. odashimae* and rice. (B) Statistical analysis of the phylogenetic tree constructed based on single-copy orthologous genes of each set of chromosomes. Histograms represent the number of each type of phylogenetic tree. (C) A phylogenetic tree is constructed using nuclear genes. The phylogenetic tree shows the B. odashimae^*^1 and *Dendrocalamus* species are closely related in the phylogenetic tree. B. odashimae*2 and B. odashimae^*^3 are closely related to *B. oldhamii*. The rice is used as an outgroup. (D) The Maximum likelihood (ML) phylogenetic tree based on complete chloroplast genomes. The phylogenetic tree revealed the genetic proximity of *B. odashimae* to *Bambusa* species by clustering in a single group. (E) Coverage (short reads of *B. oldhamii* map to *B. odashimae* genome) of each chromosome of Hap II and Hap III. The X-axis represents haplotype, and the Y-axis represents coverage. ^****^P < 0.0001.

To investigate ploidy levels and obtain a haplotype-resolved genome, we performed the genome-wide gene synteny analyses between rice (*Oryza sativa*) and *B. odashimae*, revealing a ratio of ~1:9 (Fig. 2A and Supplemental Fig. S1B). This finding prompted us to infer that it was potentially a nonaploid. In addition, *B. odashimae* exhibits a syntenic relationship with the chromosomes of *D. latiflorus* (one of the haplotypes) and *D. sinicus*, both showing a ~3:1 pattern (Supplemental Fig. S3, Supplemental Fig. S4). *Dendrocalamus latiflorus* (2n = 6x = 70) [16] and *D. sinicus* (2n = 6x = 70) [3] are hexaploids with A, B, and C subgenomes derived from different ancestors. These results indicated that, compared with these two bamboo species, the *B. odashimae* genome composes three haplotype genomes, each including A, B, and C subgenomes.

The dot plot results from NUCmer [17] revealed that within each homologous chromosome group of *B. odashimae*, one chromosome exhibited a higher similarity to *D. latiflorus* and *D. sinicus* compared to the other two chromosomes (Supplemental Fig. S3, Supplemental Fig. S4). Thus, we inferred that one parent species may be derived from *Dendrocalamus*, designating these chromosomes of *B. odashimae* as haplotype I (Hap I). To explore the possible relationships of *B. odashimae* within the *Bambusa–Dendrocalamus–Gigantochloa* complex, we constructed a chloroplast phylogenetic tree, which showed *B. odashimae* as sister to *Bambusa oldhamii* with high bootstrap support (Fig. 2D). Subsequently, we used the 75 Gb of Illumina sequencing data from the bacterial artificial chromosome (BAC) library of *B. oldhamii* and mapped the genome of *B. odashimae* to count the coverage of each chromosome. We observed significant numerical differences in the coverage of homologous chromosomes between Hap II and Hap III (Fig. 2E), with coverage differences >10% accounting for 88.2% of the cases. According to the coverage value of chromosomes (Table S9), we can distinguish the Hap II (53.5% on average ) and Hap III (35.8% on average). In addition, the collinearity results showed that the Dsi03A chromosome corresponds to only two chromosomes in *B. oldhamii* (Bod03A1 and Bod03A2), while the other 34 chromosomes of *D. sinicus* correspond to three chromosomes each in *B. oldhamii* (Supplemental Fig. S3). Therefore, 34 chromosomes are included in Hap III as a result of the loss of chromosome 3 in the A subgenome of Hap III, while Hap I and Hap II each contain 35 chromosomes.

To further reveal the relationships among the three haplotype genomes, we reconstructed 35 phylogenetic trees for each set of homologous chromosomes of woody bamboos, using rice as an outgroup, based on single-copy orthologous genes (Fig. 2B). We found that the chromosome of Hap I (Bod^*^1) and *Dendrocalamus* species formed a clade, while the chromosome of Hap II (Bod^*^2) and Hap III (Bod^*^3) were clustered separately in 32 phylogenetic trees. These findings provided further evidence that Hap I originated from *D. latiflorus* or a closely related species, whereas Hap II and Hap III had different origins.

**Figure 3:**
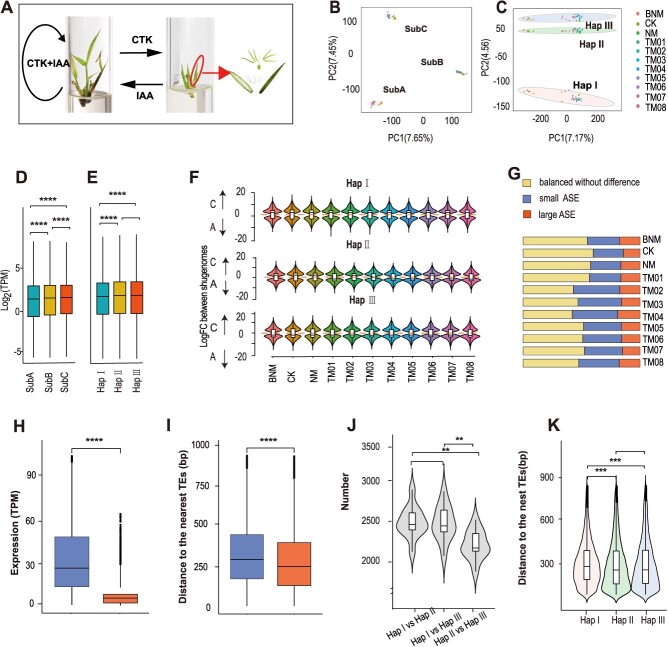
Patterns of subgenome and allelic gene expression. (A) Cytokinin (CTK)-induced *in vitro* flowering and auxin (IAA)-induced *in vitro* rejuvenation of *B. odashimae*. The pictures from left to right represent the vegetative shoots, flowering shoots, and flower anatomy of *B. odashimae*, respectively. (B) PCA of the first two principal components of subgenome expression profiles across 11 different developmental stages under different hormone treatments. (C) PCA plot of the first two principal components of allele expression profiles across 11 stages. (D) Transcript abundance across all samples for expressed genes among subgenome A, B, and C of Hap I. (E) Transcript abundance across all samples for expressed genes among Hap I, Hap II, and Hap III. (F) The violin plot of biased expression for homoeologous genes of subgenome A and subgenome C across all samples. (G) Proportions of alleles with allelic expression bias across 11 different samples. There are three categories: small allele expression differences (ASE) genes, large ASE genes, and balanced genes without difference. (H) Absolute expression abundance for the small ASE genes (left) and large ASE genes (right). (I) Distances to the nearest TEs of small ASE genes (left) and large ASE (right). (J) Analysis of the number of differentially expressed genes among different haplotype pairs. (K) Distances to the nearest TEs for large ASE in different haplotypes. The line in the center of each plot (D, E, F, H, I, J, and K) represents the median value, and the bounds of each box indicate the first (25%) and third (75%) quartiles. Mann–Whitney–Wilcoxon test. ^*^P < 0.05; ^**^P < 0.01; ^***^P < 0.001 and ^****^P < 0.0001.

Additionally, we identified 411 orthologous genes among *B. odashimae*, *B. oldhamii*, *D. latiflorus*, *D. sinicus*, *Bonia amplexicaulis*, and rice for phylogenetic inference. The analysis showed Hap I had a closer relationship to *D. latiflorus*, whereas Hap II had a closer relationship with *B. oldhamii* (Fig. 2C). The phylogenetic tree, reconstructed based on the complete chloroplast genome sequences (Fig. 2D), suggested the maternal parent of *B. odashimae* might be *B. oldhamii* or a species closely related to it. In conclusion, our results indicate that Hap I originated from *Dendrocalamus*, while Hap II and Hap III originated from *Bambusa*, with Hap II from a species closely related to *B. oldhamii* and Hap III from a more distantly related one.

### Comparative analysis of Hap I, Hap II, and Hap III

The sizes of the Hap I, Hap II, and Hap III assemblies were 1.24G, 1.06G, and 1.03G, respectively. Interestingly, we ranked each set of chromosomes according to their size and found that the majority of the chromosomes from Hap I [18] were the largest one of homologous groups (Supplemental Fig. S5). Additionally, BUSCO analysis showed that 99.3%, 93%, and 94.5% of conserved genes could be completely covered by the Hap I, Hap II, and Hap III genomes, respectively (Table S10). These results show that the Hap I genome is the largest and best assembled among the three haplotypes.

Syntenic analysis of haploid genomes reveals a high degree of synteny in different haploid genome pairs (Supplemental Fig. S6 and S7). However, our similarity analysis of allele pairs revealed that the allele pairs on Hap II versus Hap III exhibited significantly higher similarity than others (Hap I versus Hap II, Hap I versus Hap III) in 34 groups of alleles (Supplemental Fig. S8). These results show that Hap II and Hap III which are derived from the same genus, have higher similarity. In addition, compared to Hap I, 93 and 154 deletions, 111 and 149 insertions, and 22 and 29 inversions were detected in Hap II and III, respectively. This result indicates Hap III has more structural variations (SVs) than Hap II when using Hap I as a reference.

### *In vitro* flowering and rejuvenation

Nodal explants of *B. odashimae* were induced to grow on a Murashige and Skoog (MS) medium supplemented with cytokinin, resulting in *in vitro* flowering within ~5 months. Based on subculture timing, anatomical analyses, paraffin section examination, and number of leaves and shoots, the induced flowering was categorized into three stages (Supplemental Fig. S9A, S9B and S9F). The initial four generations (TM01–TM04) represented the vegetative stage, while the fifth and sixth subcultures (TM05 and TM06) marked the floral transition. The latter two subcultures (TM07 and TM08) signified the reproductive stage. Subsequent examination of spikelets revealed complete floral structures (Fig. 3A).

Auxin application induced vegetative shoot regeneration after *in vitro* flowering of *B. odashimae*. Inflorescence clusters from TM08 were treated with auxin in MS medium (NM), matured, and fell off, followed by the emergence of new vegetative shoots (Supplemental Fig. S9E), which indicated the *B. odashimae* plantlet switches back to vegetative growth. The shoots regenerated after flowering could maintain vegetative growth in MS medium without any plant growth regulator (CK) (Supplemental Fig. S9D) for over 3 years without blooming. Simultaneous addition of cytokinin and auxin in the MS medium (BNM) delayed the time of induction of flowering (Supplemental Fig. S9C). *In vitro* flowering was induced at least 6 months later and the timing of flowering varied. We collected shoots in the 11 periods/treatments using the tissue culture system. Transcriptome sequencing was subsequently performed.

### Homoeolog expression patterns

To understand subgenome expression patterns during *in vitro* flowering and rejuvenation, we identified 4299, 3223, and 3299 homoeologous gene triads (1:1:1) across the three subgenomes in Hap I, II, and III, respectively. In Hap I, compared to the A and B subgenomes, the C subgenome showed a higher proportion of expressed genes and the highest average expression level, despite having the fewest genes (Fig. 3D, Table S11). Similar results were found in Hap II and Hap III (Supplemental Fig. S10A and S10B), consistent with the findings in other sequenced hexaploid bamboos [3]. Principal component analysis (PCA) showed that the transcriptomes were mainly clustered by subgenomes and secondarily by different samples (Fig. 3B).

In each haplotype genome, most homologous genes exhibited balanced expression, while fewer showed expression bias (Table S12). However, the expression bias among the three haplotypes was inconsistent. For instance, differential expression analysis of homologous genes from the A and C subgenomes revealed that the C subgenome consistently exhibited a higher number of upregulated genes across all samples in Hap I. In contrast, Hap III demonstrated the opposite pattern (Fig. 3F). Therefore, the subgenome expression patterns in *B. odashimae* are similar to other hexaploid bamboos [3], with slight variations across different haplotypes.

### A large number of ASE genes

*Bambusa odashimae* inherits three alleles for most genes in each subgenome, from its parental genomes Hap I, II, and III. Moreover, 25 953 alleles with a 1:1:1 corresponding relationship were used to analyze allelic expression. PCA analysis showed that transcriptomes were shaped by the haplotype genome (Fig. 3C). Although Hap I, originating from the genus *Dendrocalamus*, has the largest number of genes, its expression level is significantly lower than that of Hap II and III (Fig. 3E). Additionally, as an aneuploid, chromosome 3 of the A subgenome in *B. odashimae* lacks a homologous chromosome, and its average expression level is the highest in the A subgenome (Supplemental Fig. S10C).

To explore the characteristics of allele-specific expression (ASE) genes, we divided them into three categories: (1) balanced without difference (*P* ≥ 0.05 in three pairs of alleles); (2) large ASE genes (log2 fold change (FC) > |2|, *P* < 0.05 in any pair of alleles); and (3) the remaining ones as small ASE genes (FC ≥ |2|，*P* < 0.05). During flowering and rejuvenation, the proportion of large ASE genes was the smallest (14%–19%) (Fig. 3G). Among the three allelic pairs of large ASE genes, two allelic pairs showed large ASE, accounting for 56%–59% (Table S13). Across all stages, 10 313 genes showed large ASE, accounting for 40% of the total alleles, much higher than highly heterozygous apple (19%) [19] and cultivated ginger (22.7%) [20]. The average number of ASE genes (33.90%) during flowering induction was higher than during rejuvenation induction (28.77%), suggesting greater differential expression of alleles during the flowering process. Small ASE genes exhibited higher absolute transcriptional abundance than large ASE genes (Fig. 3H), indicating that a decrease in the expression level of an allele may contribute to the large ASE genes. Furthermore, we investigated the associations of flanking transposable elements (TE) with the expression difference of alleles and found alleles in the large ASE were significantly closer to the TEs (Fig. 3I).

As Hap I and Hap II/Hap III belonged to intergeneric hybridization while Hap II and Hap III engaged in interspecific hybridization, we further analyzed the contribution of different hybrids to the number of ASE genes. The number of large ASE genes in Hap I versus Hap II/Hap III were much more than large ASE genes between Hap II and Hap III (Fig. 3J). This result suggested that intergeneric hybridization tended to produce more ASE than interspecific hybridization. We examined the distance to the TEs of ASE and found TE proximity of ASE in Hap I was significantly closer than others (Fig. 3K). In addition, the large ASE genes were also enriched in cytokinin metabolic process and regulation of hormone levels (Supplemental Fig. S10D).

### Cytokinin-related genes play an important role in inducing flowering

**Figure 4:**
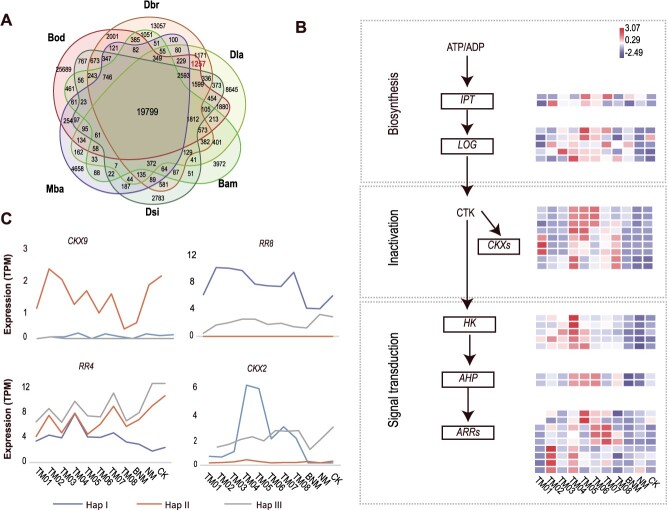
The role of cytokinin-related genes in *in vitro* flowering. (A) Venn diagram representing the number of shared and unique orthologous groups (orthogroups) among bamboo species, including *B. amplexicaulis* (Bam), *Melocanna baccifera* (Mba), *B. odashimae* (Bod), *D. latiflorus* (Dla), *D. brandisii* (Dbr), and *D. sinicus* (Dsi). (B) Heat map representing the expression of some cytokinin-related genes. (C) Expression of the alleles of the *CKX9*, *RR8*, *RR4*, and *CKX2*.

Among 12 bamboo species with published genomes [3, 14, 16], only three species, i.e. *B. odashimae, Dendrocalamus brandisii,* and *D. latiflorus* can be induced *in vitro* flowering by cytokinin [9, 11, 21]. *Bambusa odashimae, D. brandisii*, and *D. latiflorus* share 2412 (1257) unique genes (orthogroups) (Fig. 4A). The KEGG enrichment analysis of these genes revealed their enrichment in crucial pathways, such as plant hormone signal transduction (cytokinin-related genes) (Supplemental Fig. S10E and S10F). And we found cytokinin-related genes showed different types of ASE. For example, *CKX9* and *RR8* genes showed biased expression in all stages of flowering and rejuvenation, while *RR4* and *CKX2* genes exhibited allele-specific expression in certain stages (Fig. 4C). These results indicate that cytokinin-related genes are crucial for inducing flowering and that ASE significantly influences cytokinin expression.

To better explore the effect of exogenous cytokinin, we identified five cytokinin-related gene families and established expression profiles (Fig. 4B, Supplemental Fig. S11). The *Response Regulator* (*RRs*) gene family has a larger number of genes compared to the *D. sinicus* and other related species, indicating an expansion of this gene family (Table S14). In addition, we found that many genes were upregulated in the cytokinin pathway during cytokinin-induced flowering and downregulated during auxin-induced rejuvenation (Fig. 4B, and Supplemental Fig. S11 and S12).

### The *COLs* gene family links to hormone regulation of flowering and rejuvenation

**Figure 5:**
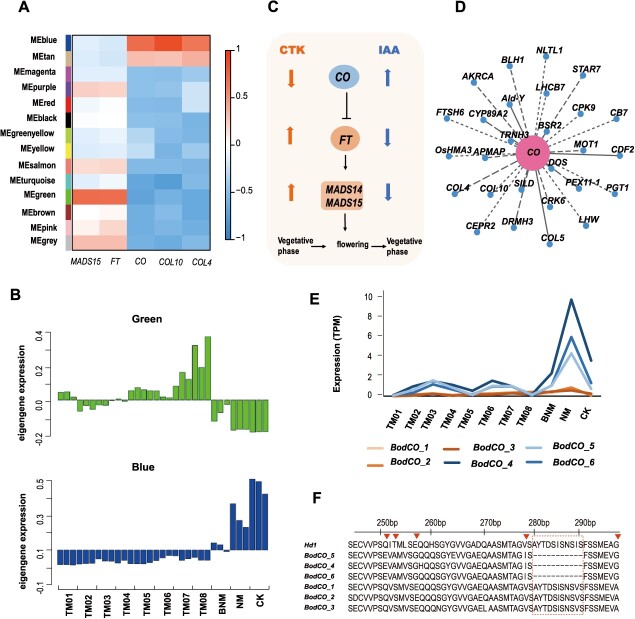
*COL* genes might regulate *in vitro* flowering. (A) Module–gene association. Each row corresponds to a module. The X-axis represents genes. (B) Eigengene expression profiles for the modules. A histogram shows the expression levels (TPM) of each module gene. The X and Y axes in the histogram indicate the value of the module eigengene and sample type. (C) Cytokinin and auxin have opposite effects on the expression of key flowering genes. The upward arrow indicates an increase in expression, and the downward arrow indicates the opposite effect. (D) Cytoscape representation of co-expressed genes with *CO*, with edge weights ≥0.15 in module ‘blue’. (E) Expression levels of six *BodCO* gene copies at 11 different stages. *BodCO_1*, *BodCO_2*, and *BodCO_3* alleles were in B subgenome, and *BodCO_4*, *BodCO_5*, and *BodCO_6* alleles were in C subgenome. The Y-axis indicates the expression levels (TPM); the X-axis indicates the sample type. (F) A global alignment of *CO* genes and alleles in rice and *B. odashimae*. The *BodCO_4*, *BodCO_5*, and *BodCO*_6 alleles were missing 11 amino acids compared to *BodCO_1*, *BodCO_2*, *BodCO_3,* and *OsCO* (*Hd1*). Red triangles indicate differences in amino acids.

We performed differential expression analysis on the different stages of *in vitro* flowering (TM01–TM08) and the vegetative shoots on MS medium (CK), identifying a total of 13 962 shared differentially expressed genes (Supplemental Fig. S13). Through the weighted gene co-expression network analysis (WGCNA) analysis, 14 WGCNA modules were identified (Fig. 5A and Supplemental Fig. S14). Our findings suggest that the ‘blue’ module may be intricately linked to hormone-mediated regulation of flowering and rejuvenation. The ‘blue’ module is markedly downregulated during cytokinin-induced flowering (TM01–TM08), significantly upregulated during rejuvenation (NM), and displays intermediate expression levels when cytokinin and auxin are administered together (BNM). Additionally, the ‘green’ module’s expression gradually increases during cytokinin-induced flowering (Fig. 5B). The ‘green’ module correlates with the expression patterns of *FLOWERING LOCUS T* (*FT*) and *MADS15* homologous genes, while the ‘blue’ module aligns closely with the expression patterns of *CO*, *COL10,* and *COL4* (Fig. 5A). This result indicates that cytokinin and auxin influence key flowering genes (Fig. 5C). The ‘blue’ module, comprising 2051 genes, is enriched in photosynthesis and the biological clock according to KEGG enrichment (Supplemental Fig. S15).

Within the ‘blue’ module, *COL4*, *COL5*, and *COL10* of the *CONSTANS-like (COL)* gene family are co-expressed with the *CO* gene (Fig. 5D). Studies have revealed that *COLs* participate in photoperiod-dependent flowering [22]. We identified 91 *COL* gene copies in the genome of *B. odashimae* and constructed a neighbor-joining tree (Supplemental Fig. S16). A heat map (Supplemental Fig. S17) showed that the expression of *COLs* was similar to *CO* genes, repressed by cytokinin and promoted by auxin. To validate the RNA sequencing (RNA-seq) findings, we examined and confirmed the expression patterns of 20 related floral transition genes by real-time reverse transcription PCR (qRT-PCR) (Supplemental Fig. S18).

The *CO* gene mediates between the circadian clock and the control of flowering [23]. We identified six CO alleles (*BodCO_1*–*BodCO_6*), with *BodCO_1*, *BodCO_2*, and *BodCO_3* in the B subgenome, while *BodCO_4*, *BodCO_5*, and *BodCO_6* in the C subgenome. Expression patterns show that *BodCO_4*, *BodCO_5*, and *BodCO_6* are strictly regulated by hormones (Fig. 5E), whereas *BodCO_1*, *BodCO_2*, and *BodCO_3* alleles do not vary significantly across different mediums. This suggests that *BodCO_4*, *BodCO_5,* and *BodCO_6* play a role in hormone regulation of flowering and regeneration, unlike *BodCO_1*, *BodCO_2,* and *BodCO_3*. We performed global sequence alignment of the protein sequences of *Heading date 1* (*Hd1*), a rice ortholog of *Arabidopsis thaliana CO*, and six *CO* alleles of *B. odashimae*. The protein sequences of the *BodCO_4*, *BodCO_5,* and *BodCO_6* alleles had a deletion of 11 amino acids compared to those of *BodCO_1*, *BodCO_2*, *BodCO_3,* and *Hd1* (Fig. 5F). This deletion might affect the structure and function of proteins. Additionally, cis-regulatory elements (CREs) of *BodCO* alleles may play a role in gene expression regulation. The CAT-box (GCCACT) is present in *BodCO_4, BodCO_5*, and *BodCO_6* but absent in *BodCO_1*, *BodCO_2*, and *BodCO_3*. Meanwhile, the light-responsive CRE (Box 4, ATTAAT), is present in *BodCO_1*, *BodCO_2*, and *BodCO_3* but absent in *BodCO_4*, *BodCO_5*, and *BodCO_6* alleles (Supplemental Fig. S19). These results indicate that CAT-box and Box 4 are potential regulatory factors, underlying the differential expression of these *CO* genes.

## Discussion

Polyploidy is widespread in the origin and evolution of woody bamboos. The haplotype-resolved genome of *B. odashimae*, a nonaploid bamboo of hybrid origin, can serve as a reference to further investigation of allopolyploidization events in bamboo. Apart from *B. odashimae*, five other PWB species have been reported to possess 104 chromosomes [5], possibly due to a similar origin pattern. The basic chromosome number for the Bambusoideae is presumably 12 [24]. Reconstruction of ancestral bamboo karyotypes (ABKs) revealed that the C subgenome in tropical woody bamboos underwent frequent chromosomal rearrangements such as fissions and fusions [3]. Based on the PWB chromosome number of 70, the combination of a 2n gamete with a 1n gamete is likely to result in nonaploidy (105). However, these nonaploid bamboos have 96, 98, 102, and 104 chromosomes and exhibit aneuploidy [5]. Recent studies suggested that triploids could produce a few normal gametes (1n, 2n, or 3n) and aneuploids were the immediate progeny, which may act as a genetic bridge in the process of allopolyploidization [25]. The nonaploid *B. odashimae* exhibits triploid characteristics in its subgenomes and is an aneuploid. Therefore, we hypothesize that nonaploids may act as a bridge in allopolyploid formation, with aneuploids as intermediates, which is a possible mechanism in the polyploidization of bamboos.

Through literature review [9, 11, 26], *in vitro* flowering of bamboos is always accompanied by hybridization. Moreover, these species have mainly been reported from the PWB genera *Bambusa* and *Dendrocalamus*, with many species of the two genera characterized by a complex evolutionary history and possibly of hybrid origin [27]. And among sequenced bamboo genomes[3, 14, 16], *B. odashimae*, *D. brandisii*, and *D. latiflorus* can be induced flowering *in vitro* [9, 11, 21] and share a high degree of heterozygosity. In addition, their shared unique genes were enriched in cytokinin-related genes. Our research revealed that hybrid *B. odashimae* has many ASE genes enriched in cytokinin-related pathway, which might affect response to exogenously applied cytokinin. And 40% of the alleles show ASE during *in vitro* flowering and rejuvenation, and the impact of ASE on gene expression is noteworthy [19]. Notably, the role of ASE genes in hybrid bamboos has long been overlooked due to the lack of haplotype genomes. Our findings establish a potential link between hybridization and the induction of flowering in *B. odashimae*, adding further value to the *in vitro* flowering system.

Woody bamboos have a unique flowering behavior, which has long been regarded as a ‘mystery’ in plants [2]. So far, there are only a few reports related to bamboo flowering [28, 29]. *Bambusa odashimae* is an excellent example for studying *in vitro* flowering in bamboos, but it has a complex genome. Through genome sequencing, we can obtain a reference for identifying genes, transcriptome analysis, and understanding the expression differences of alleles, which is helpful to unravel the molecular mechanism. We found cytokinin and auxin have opposite effects on the expression of key flowering genes, such as *COLs* genes, indicating that cytokinin promotes flowering by repressing *COLs* expression, while auxin promotes rejuvenation by enhancing *COLs* expression. It has been reported that, in nature, the expression of *CO* gene decreases during flowering in bamboos [30], as it does in *in vitro* flowering. We inferred that the long-term vegetative growth of woody bamboos might be attributed to the fact that they maintain a high level of expression of *CO* gene, thereby inhibiting the flowering process. These findings provide a valuable tool for studying the genetic and molecular mechanisms underlying flowering and vegetative regeneration in bamboos, which are challenging to investigate due to the long life cycle of bamboo plants.

In short, the *in vitro* flowering system together with a reference genome of *B. odashimae* provides an excellent opportunity for studying allopolyploidization, intergeneric and interspecific hybridization, and flowering mechanism of woody bamboos. Our study provides important insights into the ‘mystery’ of bamboo flowering and brings us closer to further gene function analysis.

## Materials and methods

### Plant materials for DNA sequencing

Fresh leaves of *B. odashimae* were collected in the greenhouse of the Germplasm Bank of Wild Species, Kunming Institute of Botany, Chinese Academy of Sciences (Kunming, Yunnan, China; N25°08′20″, E102°44′21″). DNA was extracted using the cetyltrimethylammonium bromide (CTAB) method. Next, we constructed PCR-free libraries and sequenced them on the MGISEQ-2000 platform. For ONT sequencing, high-quality DNA was extracted with the QIAGEN® Genomic kit, followed by library construction with the LSK109 kit and sequencing on the ONT platform. To position hybrid scaffolds onto the chromosome, we prepared Hi-C samples, constructed the Hi-C library, and obtained data via the Illumina Novaseq platform.

### Genome size and heterozygosity estimation

The BD FACScalibur flow cytometer was employed to detect the cellular DNA content, with tomato serving as an internal reference. Subsequently, the Modifit3.0 analysis software (https://www.vsh.com/Documentation/ModFitLT/html/installationsetup.htm) was utilized for graphical analysis. Utilizing k-mer analysis (25-mers), short reads were employed to estimate the genome size and heterozygosity using Jellyfish v.2.3.0 [31] and GenomeScope2 [32].

### *De novo* assembly

Using the sequenced data, 105Gb of short paired-end reads, 176 Gb of ONT long reads, and 352Gb Hi-C paired-end reads, we constructed a *de novo* assembly of *B. odashimae.* After quality control, ONT long reads underwent self-correction to produce consistent sequences (CNS reads) and were then assembled into the genome using NextDenovo v2.3.1 (https://github.com/Nextomics/NextDenovo) software with parameters reads_cutoff: 1 k and seed_cutoff: 31 k. Then, to enhance assembly accuracy, the short paired-end reads and ONT reads were mapped to the preliminary genome, and the preliminary genome was corrected by Nextpolish v1.3.0 [33], resulting in a 3.36 Gb polished genome.

To examine the completeness of the assembly, we used BUSCO v4.0.5 [34], CEGMA v2 [35], BWA [36], and SAMtools v0.1.1855 [37]. The coverage of expressed genes of the *B. odashimae* genome was examined by aligning all the RNA-seq reads against the assembly using HISAT2 v2.1.0 with default parameters [38].

### Annotation of the genome

TE in the *B. odashimae* genome were found using a combination of *de novo* and homology-based methods, and the results indicated that 68.18% of the genome consists of TE. Briefly, a *de novo* repeat library for *B. odashimae* was predicted using MITE-hunter [39], RepeatModeler v1.0.11, LTR_FINDER v1.07 [40], LTRharvest v1.6.5 [41], and LTR_retriver v2.9.0 [42]. RepeatMasker v1.331 [43] was then applied to identify known and novel TEs using homology-based method.

Three independent approaches, including homolog prediction, *de novo* prediction, and transcriptome-assisted prediction, were employed for gene prediction. GeMoMa v1.6.1 [44] was used for the homology search,. Three tools, including the STAR v2.7.3 [45], PASA v2.3.3 [46], and Stringtie2 v2.0.6 [47] software, were employed for RNAseq-based gene prediction. *De novo* prediction utilized Augustus v3.3.1 [48] with default parameters. Finally, an integrated gene set was obtained using EVM v1.1.1 [46].

### Hi-C analysis

Firstly, we aligned the paired-end reads to the genome using bowtie2 v2.3.2 (−end-to-end—very-sensitive -L 30) [49], obtaining localization information and uniquely mapped paired-end reads. Secondly, HiC-Pro v2.8.1 [50] was used to identify and retain valid interaction paired reads. Thirdly, the contigs were further clustered by LACHESIS [51], with parameters CLUSTER_MIN_RE_SITES = 100, CLUSTER_MAX_LINK_DENSITY = 2.5, ORDER MIN N RES IN TRUNK = 60, ORDER MIN N RES IN SHREDS = 60, CLUSTER NONINFORMATIVE RATIO = 1.4. After LACHESIS clustering, a total of 3606.11 Mb sequences were mapped to 104 chromosomes, accounting for 99.97% of the total length. Finally, obvious placement and orientation errors were manually adjusted, and the chromosome mounting rate was 99.50%.

### Haplome construction

We first aligned the genome of *B. odashimae* against the genomes of *D. sinicus* and *D. latiflorus* using NUCmer in MUMMER4 [17] with the parameters of ‘-i 97 -l 8000’. Genome-wide gene synteny analyses showed a 1:3 ratio between *D. sinicus* and *B. odashimae*, and the same result between *D. latiflorus* (haploid genomes) and *B. odashimae*. Therefore, we can construct the three haplomes (haploid genomes) and obtain 35 sets of chromosomes, each comprising three chromosomes with distinct alleles, except for Bod03A. We observed that within these 35 sets of chromosomes, there consistently existed one chromosome whose similarity to *D. sinicus* and *D. latiflorus* was markedly higher than the other two. This difference was shown in the dot plots from the NUCmer output. Therefore, these chromosomes were selected to form Hap I.

Because *B. oldhamii* and *B. odashimae* have similar morphology and are closely related, we used *B. oldhamii* to help distinguish Hap II and III of *B. odashimae*. The 75 Gb of Illumina sequencing data from the BAC library of *B. oldhamii* (SCIENCE DATA BANK:10.57760/sciencedb.15372) were downloaded and mapped to the genome of *B. odashimae* using BWA-MEM v0.7.17 [52] with default settings. The bamdst v1.0.6 (https://github.com/shiquan/bamdst) was then used to count the coverage of each chromosome. Finally, Hap II and III were distinguished by the size of the coverage of homologous chromosomes. Hap II had a larger coverage value, while Hap III had a smaller one.

To assess the accuracy of haploid genome classification, we constructed phylogenetic trees at both the chromosome and genome levels. We downloaded the protein sequences of *D. sinicus* [15], *D. latiflorus* [16], *B. amplexicaulis* (https://bamboo.genobank.org) [15], *B. oldhamii* (SCIENCE DATA BANK: 10.57760/sciencedb.09848), and rice (The Rice Annotation Project Database). To construct phylogenetic trees at the chromosome level, we first divided the homologous chromosomes of *B. odashimae*, *D. sinicus*, *D. latiflorus*, and *B. amplexicaulis* into 35 groups. Next, we employed OrthoFinder v2.5.5 [53] to find single-copy orthologous genes and further divided them into 35 groups based on homologous chromosomes. Finally, we constructed 35 maximum likelihood phylogenies using IQ-TREE v2.0.3 [54], employing the single-copy orthologous genes. In addition, to construct phylogenetic trees at the genome level, we identified orthologous genes among *B. odashimae*, *B. oldhamii*, *D. latiflorus*, *D. sinicus*, *B. amplexicaulis*, and used rice as an outgroup for phylogenetic inference. Afterward, we employed a technique akin to the previously outlined method to build the phylogenetic tree. Additionally, we used the default parameters of the MUM&Co software [55] to detect structural variations between haplotypes.

### Chloroplast phylogenetic tree reconstruction

Based on previous studies [27, 56], we downloaded the complete chloroplast genome sequences of relevant species. The chloroplast genomes were aligned by the software MAFFT v7.475 [57]. And the aligned sequences were then trimmed by trimAL v1.4.rev6 [58]. Finally, the chloroplast phylogenetic tree was built using IQ-TREE v2.0.3 [54].

### Identification of alleles and homoeologous genes

In allopolyploidy, homoeologous genes are ‘subgenome orthologs’, originate from different ancestors, forming subgenomes. Alleles, refer to alternative forms of genes at a given site on homologous chromosomes. As a PWB, *B. odashimae* possesses A, B, and C subgenomes. Each genetic locus on these subgenomes has three alleles.

The identification of synteny blocks between two haplotypes (Hap I versus Hap II, Hap II versus Hap III, and Hap I versus Hap III) was conducted employing MCScanX (Python version) [59], with specific parameters (-no_strip_names and -cscore = 0.99). Then, collinear blocks that probably resulted from whole-genome duplication were manually removed. Finally, we combined these results to identify allele gene sets (Hap I:Hap II:Hap III = 1:1:1) in each subgenome. MCScanX software (default parameters) was also used to identify homoeologous gene groups among A, B, and C subgenomes, in each haplotype genome, respectively. The steps were consistent with the identification of the alleles, resulting in the identification of homoeologous genes with a 1:1:1 ratio.

### Similarity analysis of allele pairs

We identified a total of 25 779 alleles with a 1:1:1 ratio and categorized them into 34 groups corresponding to allelic chromosomes. We then calculated the coding sequence of alleles pair similarity by Identify v1.2 software [60]. Finally, the similarities were statistically analyzed and plotted.

### Analyze expression of homologous genes

After RNA extraction for all samples and construction of the RNA-Seq library, the transcriptome was sequenced. The clean reads were mapped to the assembled *B. odashimae* genome using HISAT2 v2.1.0 [38] with default parameters. The featureCounts v2.0.0 [18] was employed for the quantification of gene expression. And the DESeq2 v3.0 [61] was used to find differentially expressed genes (DEGs), and thresholds for the identification of DEGs were a fold change of TPM > 2 and FDR < 0.01.

To elucidate subgenome expression bias, we initially conducted a differential analysis for each subgenome pair (A versus B, A versus C, and B versus C) in three haplotypes, respectively. Subsequently, we employed a methodology similar to that used in wheat studies [62] to further investigate homoeolog expression bias in each sample.

For each subgenome, most genes of *B. odashimae* have three alleles, so the analysis of ASE genes should first be divided into three pairs of alleles (Hap I versus Hap II, Hap II versus Hap III, and Hap I versus Hap III) and then use DEseq2 software to analyze the differential alleles, and finally make statistics. ASE can be divided into three categories, and the classification method has been described in the previous section.

### The distance between the flanking TE and the ASE gene

The Bedtools v2.25.0 software [63] was used to calculate the distance between the flanking TE and the ASE gene with parameters: -io，-d. The results were plotted and statistically performed through the R package ‘ggplot2’.

### Gene co-expression network

The co-expression network analysis of DEGs was constructed with the R package ‘WGCNA’ [64]. The soft power threshold was 14. The modules were obtained with the blockwiseModules function using TOMType = ‘unsigned’, the minimum module size was set to 30, deepSplit = 2, and mergeCutHeight = 0.22.

### Tissue culture

For details about methods of tissue culture and plant materials for transcriptome, see Supplementary material.

### The gene family and expression analysis

To search protein sequences of the related gene families, we used the HMMER v3.2.1 [65] and the BLAST (−evalue 1e-10). Use the MAFFT v7.475 [57] to align all the protein sequences (including sequences of *A. thaliana* and rice) with the parameter, —maxiterate 1000 —globalpair. The MEGA 11.0 software [66] was used to construct a Neighbor-Joining tree with the parameter setting: Bootstrap method is 1000, and substitution models is Poisson model. Then use the FigTree v1.4.4 software [67] to beautify the tree. Using the R package ‘pheatmap’, a heat map of gene expression levels was drawn.

### Real-time RT-qPCR analysis

Total RNA was reverse transcribed to cDNAs using the TransScript One-step gDNA Removal and cDNA Synthesis SuperMix (TransGen Biotech, China). TransStart Tip Green qPCR SuperMix TransGen Biotech, China, was used to measure the expression level of the targeted genes. Relative fold change in gene expression was estimated following the 2-ΔΔCt method, with UBC serving as the reference gene. Primers for qRT-PCR analysis were designed by Primer Premier 6 (Table S15).

## Supplementary Material

Web_Material_uhae250
