# Supplementary material for: Haplotype-resolved nonaploid genome provides insights into in vitro flowering in bamboos
Source: Hortic Res. 2024 Sep 4;11(12):uhae250. doi: 10.1093/hr/uhae250 (PMC11630085; doi:10.1093/hr/uhae250)
Supplement: Web_Material_uhae250 [file web_material_uhae250.zip › Supplementary_reviewed.pdf]

Supplementary information

Here is an additional note on the method and Tables S1-S15.

Methods of tissue culture

Shoot tips of the nodal explants were induced flowering in MS medium containing 0.1 mg/L TDZ (TMs). In this process, vegetative shoots emerged first, and later inflorescences gradually emerged. We collected 50-80 explants for *in vitro* flower induction every 21 days, and repeated eight times. After repeating eight times, we obtained *in vitro* shoots at different stages, including the vegetative stages (TM01-TM04), the stages of floral transition (TM05 and TM06), and the reproductive stages (TM07 and TM08). *In vitro* grown inflorescence clusters of *B. odashimae* (TM08) were transferred to MS medium containing 5 mg/L of NAA, and the vegetative shoots emerged. Continuously subcultured in this medium, floral tissue gradually disappeared and only vegetative tissue continued to grow, which means a transition from reproductive growth to vegetative growth. Placing vegetative shoots (TM04) in MS medium supplemented with auxin and cytokinin (2 mg/L 6- BenzylAdenine and 0.2 mg/L NAA) delayed flowering. Vegetative shoots of inflorescence origin could maintain vegetative growth in the MS medium without any plant growth regulators (CK) and had not bloomed for more than three years. Except for the plant growth regulators contained in the medium, the rest of the culture conditions were the same. All media contained 3% sucrose and cultures were kept under artificial light with a light/dark cycle of 16/8 h at 26°C. Cultures were subcultured to fresh medium every three weeks. In addition, the shoots with low proliferation rates were manually eliminated during the subculture process to improve the consistency of growth and development stages and shorten the flowering time.

Plant materials for transcriptome

We collected plant materials at different stages, TM01 (2d), TM02 (21d), TM03 (42d), TM04 (63d), TM05 (84d), TM06 (105d), TM07 (126d), TM08 (147d), BNM (126d), CK (126d), and NM (180d). The shoot tips with leaf primordia from TM01, the inflorescences from TM08, and leaves from others (TM02~TM07, BNM, NM, CK) were sampled on the same day, and the sampling was repeated 3-5 times. The selection of plant materials required consistent growth and development, meaning the number of leaves, the number of lateral buds, and the growth state needed to be uniform. Any materials that developed too early or too late were removed. The time of sampling was at the 14th hour of the light. After sample collection, we immediately placed into liquid nitrogen for storage and then transferred it to a -80°C refrigerator for storage.

Table S1. Cellular DNA content was measured by flow cytometry.

Sample	Reference	Fluorescence intensity	Ratio	Genome content (Gb)
L1	Tomato	90.27	2.06	3.62
L2	Tomato	91.40	2.07	3.64
L3	Tomato	94.88	2.06	3.62
L4	Tomato	90.06	1.92	3.38
L5	Tomato	85.53	1.98	3.50

Table S2. Statistics of contig for genome assembly of *B. odashimae*.

**Stat Type**	**Contig Length(bp)**	**Contig Number**
N50	15,528,522	78
N60	13,144,913	103
N70	10,497,378	134
N80	7,478,637	174
N90	4,108,276	241
Longest	49,479,938	1
Total	3,608,508,007	611
Length>=1kb	3,608,508,007	611
Length>=2kb	3,608,508,007	611
Length>=5kb	3,608,508,007	611

Table S3. Summary of chromosome size.

The set of chromosomes	Chromosome size (Mb) in haplotype Ⅰ	Chromosome size (Mb) in haplotype Ⅱ	Chromosome size (Mb) in haplotype Ⅲ
Bod01A	61.68	55.36	55.99
Bod01B	60.15	55.19	56.62
Bod01C	41.18	38.27	34.95
Bod02A	41.77	28.74	39.57
Bod02B	47.20	43.29	36.16
Bod02C	35.31	31.01	30.51
Bod03A	53.00	44.37	
Bod03B	53.47	53.37	49.01
Bod03C	32.66	31.83	22.72
Bod04A	48.34	29.18	50.33
Bod04B	36.37	34.94	26.55
Bod04C	39.29	23.46	33.91
Bod05A	31.97	26.49	30.82
Bod05B	33.40	28.38	23.60
Bod05C	27.96	26.33	26.17
Bod06A	37.09	38.30	29.42
Bod06B	35.82	31.97	35.73
Bod06C	38.44	16.89	35.01
Bod07A	31.73	29.32	28.43
Bod07B	34.46	30.27	28.78
Bod07C	25.95	24.85	24.00
Bod08A	37.43	36.55	21.13
Bod08B	31.38	17.65	29.17
Bod08C	25.87	21.31	26.95
Bod09A	36.39	30.35	33.10
Bod09B	25.20	22.84	16.24
Bod09C	30.45	24.07	29.04
Bod10A	24.18	23.95	18.02
Bod10B	27.71	25.03	19.33
Bod10C	24.89	24.81	19.97
Bod11A	58.46	47.56	56.08
Bod11B	28.51	26.67	27.29
Bod11C	26.27	25.42	20.22
Bod12A	23.85	16.43	22.14
Bod12B	25.09	25.62	22.81

Table S4. Statistics for gene function annotation.

Type	Number	Percent (%)
Swissprot	116,803	77.97
KEGG	51,489	34.37
KOG	78,433	52.36
GO	83,758	55.91
NR	141,072	94.17
Total Annotated	141,732	94.61

Table S5. Statistical results of TE repeats.

Super family	Number of elements	Length of sequence (bp)	Percentage of sequence (%)
Copia	1749553	591247443	16.39
Ale	146251	55028179	1.53
Gypsy	1284980	648347354	17.97
Bianca	61471	37550641	1.04
Sire	98355	52777452	1.46
Tork	26468	11923900	0.33
Non-chromovirus	99437	53706922	1.49
Chromovirus	83691	32290581	0.9
Ikeros	34386	16028077	0.44
Tar	33643	13216171	0.37
Ivana	64239	22264317	0.62
Angela	14158	10156293	0.28
SINE	45000	5469446	0.15
MuLE-MuDR	61402	21489574	0.6
PIF-Harbinger	60935	12262728	0.34
TcMar-Stowaway	25732	3715587	0.1
MULE-MuDR	144170	36835574	1.02
hAT-Tip100	27916	11137294	0.31
hAT-Ac	49719	15068342	0.42
hAT-Tag1	17047	4648007	0.13
CMC-EnSpm	278610	119805526	3.32
MITE	192684	38765059	1.07
Helitron	63759	12630040	0.35
Total LTR	5487046	1990635155	55.18
Total TEs	7315845	2459307133	68.18
Total Repeats	8055755	2544521480	70.54

Table S6. BUSCO evaluation of genome assemblies.

Type	Number	Percent
Complete BUSCOs (C)	1,588	98.39%
Complete and single-copy BUSCOs (S)	224	13.88%
Complete and duplicated BUSCOs (D)	1,364	84.51%
Fragmented BUSCOs (F)	3	0.19%
Missing BUSCOs (M)	23	1.43%
Total BUSCO groups searched	1,614	100%

Table S7. CEGMA analysis of *B. odashimae*.

	complete		complete + partial	
	**Prots**	**%completeness**	**Prots**	**%completeness**
Total	226	91.13	240	96.77
Group1	58	87.88	64	96.97
Group2	52	92.86	54	96.43
Group3	54	88.52	60	98.36
Group4	62	95.38	62	95.38

Table S8. The mapping rate of expressed genes in *B. odashimae* transcripts.

name	mapping rate (%)
BNM_rp01	91.90
BNM_rp02	93.20
BNM_rp03	91.23
CK_rp01	95.60
CK_rp02	96.08
CK_rp03	95.92
NM_rp01	92.47
NM_rp02	92.39
NM_rp03	91.26
TM01_rp01	92.82
TM01_rp02	92.46
TM01_rp03	90.61
TM02_rp01	96.27
TM02_rp02	94.73
TM02_rp03	96.72
TM03_rp01	96.64
TM03_rp02	95.71
TM03_rp03	96.80
TM04_rp01	96.20
TM04_rp02	96.83
TM04_rp03	96.66
TM05_rp01	96.50
TM05_rp02	96.40
TM05_rp03	96.53
TM06_rp01	96.71
TM06_rp02	96.39
TM06_rp03	96.70
TM07_rp01	96.91
TM07_rp02	96.88
TM07_rp03	96.86
TM08_rp01	97.30
TM08_rp02	96.94
TM08_rp03	96.97

Table S9. The coverage value of chromosome.

Homologous chromosome group	HapII (%)	HapIII (%)	Coverage difference value
Bod01A	49.59	31.08	18.51
Bod01B	50	27.61	22.39
Bod01C	44	34.73	9.27
Bod02A	54.61	39.05	15.56
Bod02B	47.99	30.67	17.32
Bod02C	50.36	37.39	12.97
Bod03B	56.27	36.09	20.18
Bod03C	55.4	34.64	20.76
Bod04A	49.57	42.8	6.77
Bod04B	56.24	39.45	16.79
Bod04C	56.98	42.57	14.41
Bod05A	53.75	35.7	18.05
Bod05B	56.87	30.2	26.67
Bod05C	58.07	34.64	23.43
Bod06A	52.68	29.12	23.56
Bod06B	59.12	34.32	24.8
Bod06C	60.87	44.24	16.63
Bod07A	53.38	38.13	15.25
Bod07B	53.96	38.34	15.62
Bod07C	40.13	37.56	2.57
Bod08A	49.39	35.31	14.08
Bod08B	57.75	35.54	22.21
Bod08C	58.9	37.78	21.12
Bod09A	48.91	37.74	11.17
Bod09B	50.46	30.69	19.77
Bod09C	53.26	30.46	22.8
Bod10A	58.88	38.18	20.7
Bod10B	54.78	33.63	21.15
Bod10C	57.27	35.27	22
Bod11A	50.52	42.59	7.93
Bod11B	52.44	33.44	19
Bod11C	55.17	31.74	23.43
Bod12A	57.99	42.48	15.51
Bod12B	53.37	32.47	20.9

Table S10. BUSCOs analysis of haplotype genomes of *B. odashimae*.

	Complete BUSCOs	Complete and single-copy BUSCOs	Complete and duplicated BUSCOs	Fragmented BUSCOs	Missing BUSCOs
Percent (%) of haplotype I	99.0	37.3	62.0	0.4	0.3
Percent (%) of haplotype II	97.2	34.5	62.7	0.8	2.0
Percent (%) of haplotype III	87.1	54.1	32.9	4.7	8.3

TableS11. Gene expression levels in each haplotype genome.

		Number of genes	Number of expressed genes	Proportion of expressed genes
Hap Ⅰ	AⅠ	20386	18597	0.912243697
	BⅠ	17630	16069	0.911457742
	CⅠ	16390	15196	0.927150702
HAPⅡ	AⅡ	16765	15416	0.919534745
	BⅡ	17151	15726	0.916914466
	CⅡ	13366	12446	0.931168637
Hap Ⅲ	AⅢ	16577	15081	0.909754479
	BⅢ	15385	14020	0.911277218
	CⅢ	15230	14252	0.935784636

The expressed genes were counted requiring TPM ≥ 1 in at least two samples.

Table S12. Subgenomic expression patterns in Hap Ⅰ, HapⅡ, and Hap Ⅲ.

HAP Ⅰ	Balanced	Dominant			Suppressed		
		A	B	C	A	B	C
BNM	60.92%	2.43%	2.48%	1.65%	10.92%	10.44%	11.17%
CK	52.91%	3.88%	3.50%	3.50%	11.89%	11.99%	12.33%
NM	52.38%	3.59%	3.16%	2.43%	12.33%	13.20%	12.91%
TM01	56.65%	3.06%	2.23%	2.09%	10.49%	11.89%	13.59%
TM02	67.33%	1.46%	1.36%	1.55%	8.59%	9.37%	10.34%
TM03	64.08%	1.89%	1.65%	1.46%	9.51%	10.24%	11.17%
TM04	65.92%	2.14%	1.36%	1.31%	8.25%	10%	11.02%
TM05	58.64%	3.45%	2.52%	2.43%	8.98%	11.12%	12.86%
TM06	65.97%	2.62%	1.36%	1.26%	8.40%	10.19%	10.19%
TM07	64.71%	2.04%	2.14%	1.46%	9.76%	10.05%	9.85%
TM08	61.17%	1.99%	2.14%	1.84%	12.23%	9.56%	11.07%
Average	60.97%	2.60%	2.17%	1.91%	10.12%	10.73%	11.50%
Combined analysis	60.97%	2.59%	2.17%	1.91%	10.12%	10.73%	11.50%

HAP Ⅱ	Balanced	Dominant			Suppressed		
		A	B	C	A	B	C
BNM	58.35%	3.09%	2.21%	2.95%	11%	9.93%	13.08%
CK	49.70%	3.29%	3.89%	3.22%	13.35%	12.61%	14.15%
NM	50.57%	3.76%	3.35%	2.88%	12.01%	12.01%	15.90%
TM01	53.12%	1.88%	2.88%	2.82%	11.67%	12.14%	13.62%
TM02	63.58%	1.88%	1.68%	1.54%	9.66%	10.19%	11.47%
TM03	61.10%	2.28%	1.74%	1.48%	10.73%	10.46%	12.61%
TM04	63.65%	3.55%	1.81%	1.14%	9.39%	9.79%	11.94%
TM05	53.99%	2.48%	3.02%	2.35%	10.66%	11.80%	14.62%
TM06	62.11%	2.41%	1.81%	1.14%	10.80%	9.79%	11.87%
TM07	60.83%	2.21%	1.95%	2.01%	9.93%	10.26%	12.61%
TM08	58.62%	2.68%	2.15%	1.81%	10.46%	11.80%	12.94%
Average	57.78%	2.68%	2.41%	2.12%	10.88%	10.98%	13.16%
Combined analysis	57.78%	2.66%	2.41%	2.12%	10.88%	10.98%	13.16%

HAP Ⅲ	Balanced	Dominant			Suppressed		
		A	B	C	A	B	C
BNM	57.55%	4.16%	1.74%	3.09%	9.59%	14.22%	9.66%
CK	47.48%	4.69%	2.68%	4.36%	11.33%	16.63%	12.81%
NM	50.91%	4.56%	3.09%	3.82%	11.07%	13.95%	12.61%
TM01	52.38%	4.56%	2.21%	3.69%	10.53%	15.09%	11.54%
TM02	62.11%	2.68%	1.21%	1.88%	8.72%	12.94%	10.46%
TM03	61.84%	3.35%	1.34%	2.08%	7.91%	13.62%	9.86%
TM04	62.58%	3.22%	1.01%	1.81%	8.79%	12.94%	9.66%
TM05	55.67%	3.22%	2.28%	2.75%	10.06%	13.75%	11.40%
TM06	63.98%	3.22%	1.27%	1.95%	8.92%	11.60%	9.26%
TM07	62.64%	3.22%	1.41%	2.15%	8.12%	12.68%	9.73%
TM08	60.16%	3.22%	1.61%	2.28%	8.58%	12.94%	10.93%
Average	57.94%	3.65%	1.80%	2.71%	9.42%	13.67%	10.72%
Combined analysis	57.94%	3.74%	1.80%	2.71%	9.42%	13.67%	10.72%

Table S13. The number and proportion of ASE exhibited by different numbers of allele pairs *in vitro* flowering and rejuvenation of *B. odashimae*.

	One allelic pair showed ASE	Two allelic pairs showed ASE	Three allelic pairs showed ASE
TM01	1885 (49.0 %)	2563 (55.62 %)	160 (3.47 %)
TM02	1471 (37.37 %)	2319 (58.91 %)	146 (3.71%)
TM03	1537 (37.98 %)	2339 (57.81 %)	170 (4.20 %)
TM04	1538 (37.64 %)	2378 (58.19 %)	170 (4.16 %)
TM05	1886 (38.32 %)	2821 (57.32 %)	214 (4.34 %)
TM06	1572 (37.81 %)	2415 (58.09 %)	170 (4.08 %)
TM07	1628 (36.49 %)	2624 (58.82 %)	209 (4.68 %)
TM08	1634 (38.61%)	2443 (57.74 %)	154 (3.64 %)
NM	1737 (41.05 %)	2372 (56.06 %)	122 (2.88 %)
BNM	1543 (41.92 %)	2031 (55.19 %)	106 (2.88 %)
CK	1694 (37.30 %)	2674 (58.88 %)	173 (3.81 %)

Table S14. The cytokinin-related gene families.

	Genes from each genome						Alleles of Bod			
	Osa	Zma	Sbi	Rgu	Dsi	Bod	Hap Ⅰ	Hap Ⅱ	Hap Ⅲ	all alleles
CKX	36	35	36	34	57	60	49	48	48	145
RRs	40	50	39	56	84	90	71	63	62	196
HK	6	7	3	4	13	14	8	12	9	29
Hp	5	5	5	5	11	13	9	8	6	23
IPT	10	11	9	11	18	19	19	16	15	50

Osa, rice; Zma, maize; Sbi, Sorghum; Rgu, *Raddia guianensis*; Dsi, *Dendrocalamus sinicus*.

Table S15. Primers for qRT-PCR.

name	primer1	name	primer2
BodMADS50-F	AATCAGCTTACGTTGGCGACATCTT	BodMADS50-R	AGCGATGTCTGCCAGGTAATCCA
BodMADS56-F	GAAGAGCCTCCACAACATCAGAAGA	BodMADS56-R	AGCAGTGTCCTCTCCTTCTCTTTCA
BodMADS22-F	GGAGAACATGCAACTGAGGAACCAA	BodMADS22-R	GCAGACATCACAGATTCAGAGGACT
BodSPL8-F	TTCGTCGTCTGGGTCGTGCTT	BodSPL8-R	TGTGCTGGTGGTGCTGATGGA
BodSPL6-F	GCTGAACAGCTCACCGTCTTCC	BodSPL6-R	TCGTCGTCGTCGTCGTCATCA
BodGI-F	TGAATCCTGTGCCTCAATGAACCTT	BodGI-R	GTCCTCAGTGACCTCCTCAGAAGAA
BodGI.2-F	GCACTCAGCATGTCCGTCCTTC	BodGI.2-R	ATCCGCTTGCCAGTTGATGATGTT
BodDof-F	GAGACGACAAGAACCAGAGCAACAA	BodDof-R	CGGAGGTGTAGTAGGCAGGAGATG
BodMADS1-F	GAGATACCGCACCTGCAACAACA	BodMADS1-R	ACTCAACTCTGGTCTTCAGCTTCAA
BodCOL10-F	GACGACGAGAAGCCTCAAGTGAAC	BodCOL10-R	TCACGGGTCCACGAGGAGATGA
BodCOL3-F	CGCTCGATGGACGACATCAAGG	BodCOL3-R	CCGACGATGATACGCTGTGGTTC
BeUBC-F	CCAGTGGCATGGCTGACTCA	BeUBC-R	ACGCAAGACAAGACAGGCAAGA
BodFT-F	CGTTTGTACACCATTGCACTTGGT	BodFT-R	TCCATCCATCCATCCATCAATGC
BodFT2-F	GCCGTTTGTACACCATTGCAC	BodFT2-R	TGGAGTTCACATTCTTCTGCCAC
BodCOL4-F	CACAGATCAGCGGACACTC	BodCOL4-R	GCAGCCATTGCATGGATGC
BodMADS50.2-F	GGGAGAGGAGAGGAGACTTGC	BodMADS50.2-R	GCATGCAGACAGCAGAGAGC
BodRR9-F	CAAGGAGCACTACCACCAGGAAC	BodRR9-R	TGCGGCTGTTGCTGCTATCG
BodCKX1-F	CGTGGTGTCGCTGCTCTTCT	BodCKX1-R	GTAATGCGGCAGGTACTCCTTGTA
BodRR2-F	TCGGATCACATCGCCAAGAAGG	BodRR2-R	CGAGCAGGATGAACTTGAAGAGGA
BodGID-F	CAACTTCAAGGTGGCGTACAACAT	BodGID-R	TGACGTGGTCGAACGAGGAGA
