# Supplementary figures and images for: Haplotype-resolved nonaploid genome provides insights into in vitro flowering in bamboos
Source: Hortic Res. 2024 Sep 4;11(12):uhae250. doi: 10.1093/hr/uhae250 (PMC11630085; doi:10.1093/hr/uhae250)

A

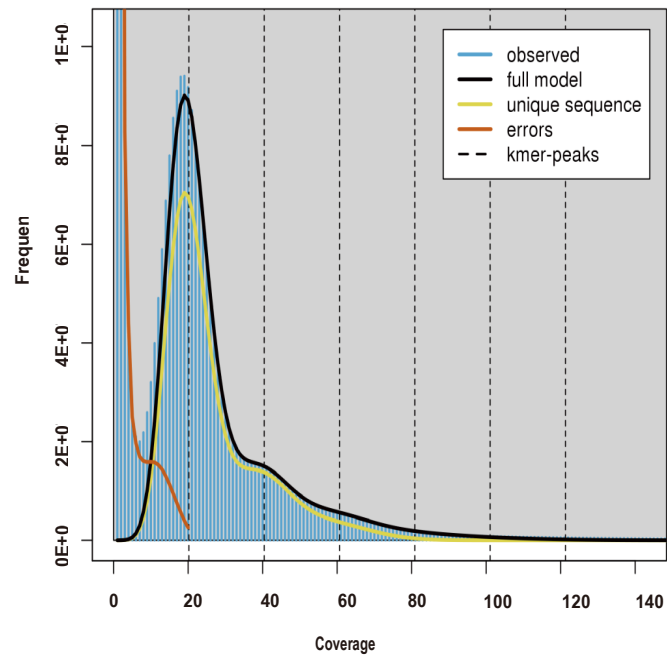

B

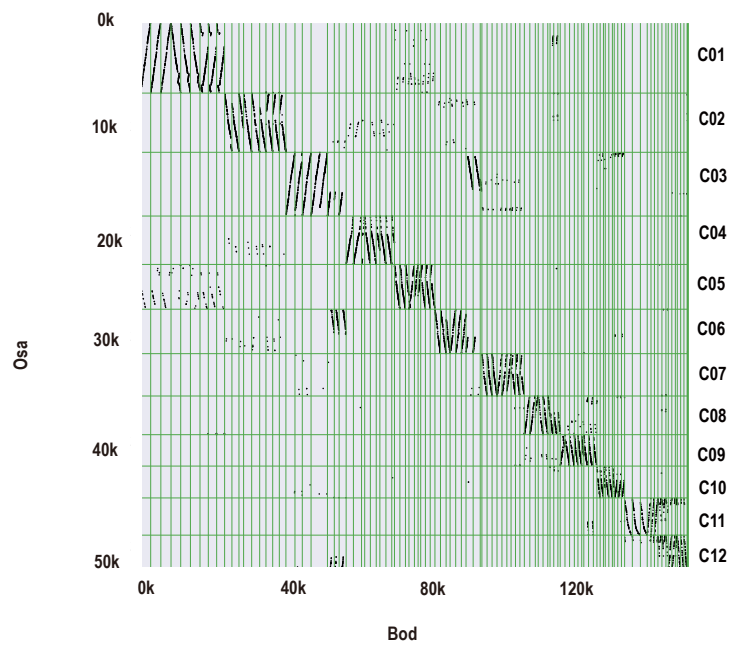

Supplement: Web_Material_uhae250 [file web_material_uhae250.zip › SFigure_1.pdf]

**A**

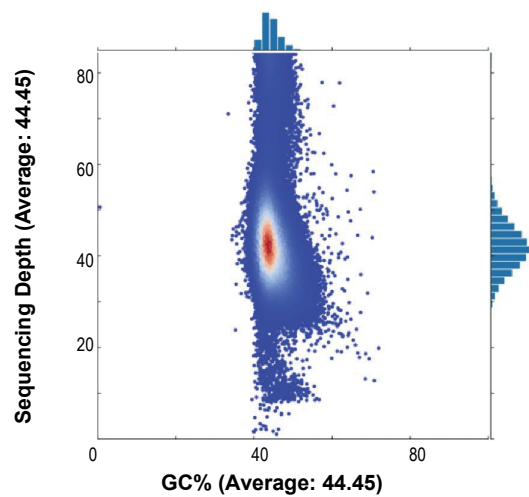

**B**

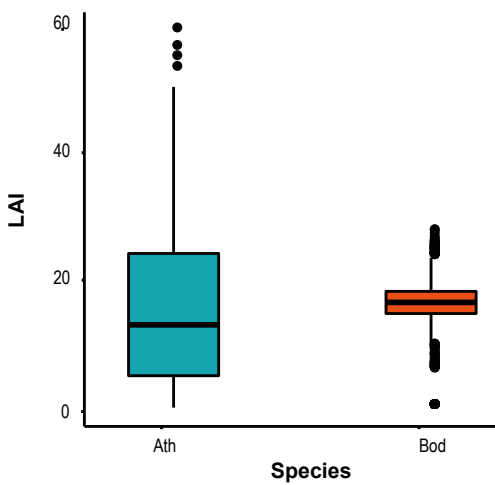

Supplement: Web_Material_uhae250 [file web_material_uhae250.zip › SFigure_2.pdf]

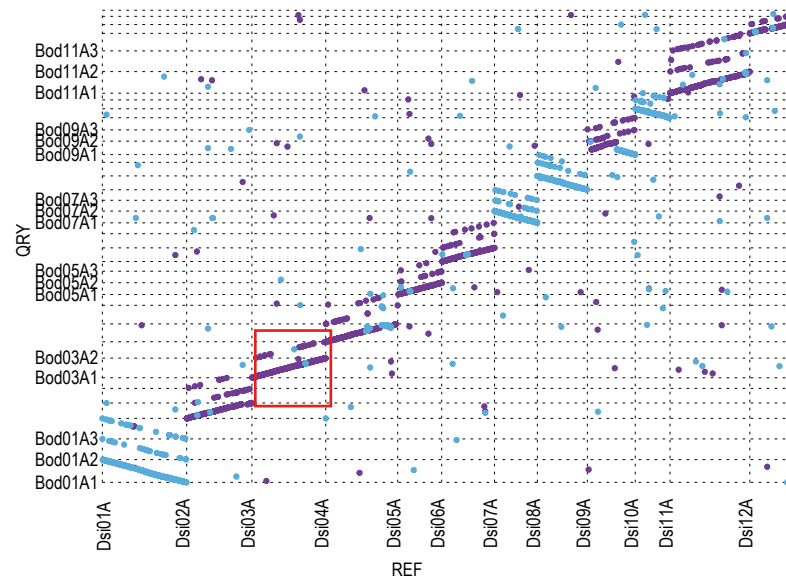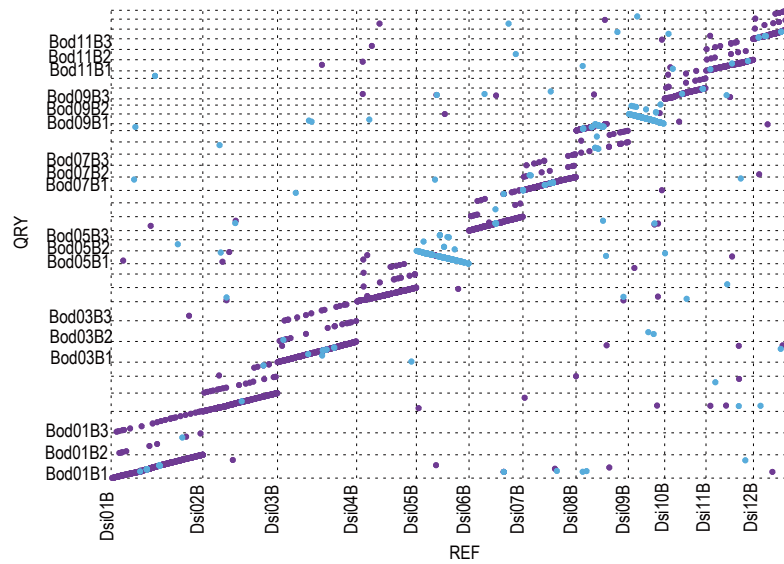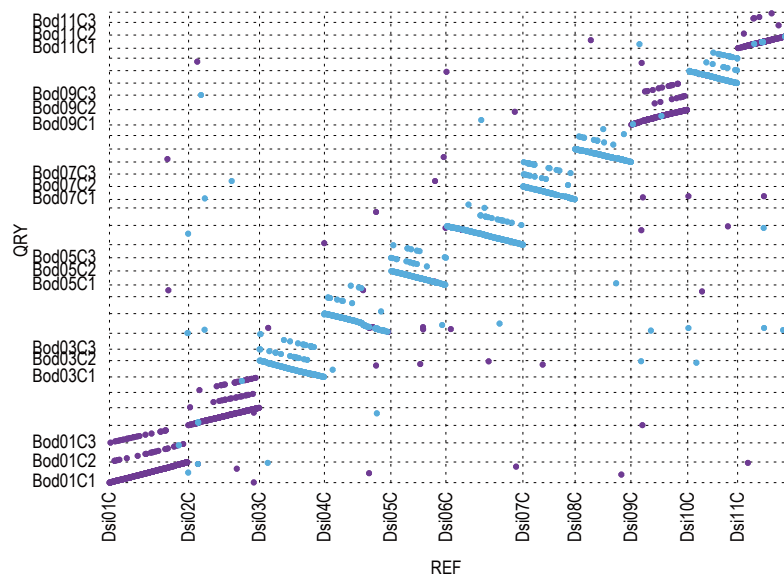

Supplement: Web_Material_uhae250 [file web_material_uhae250.zip › SFigure_3.pdf]

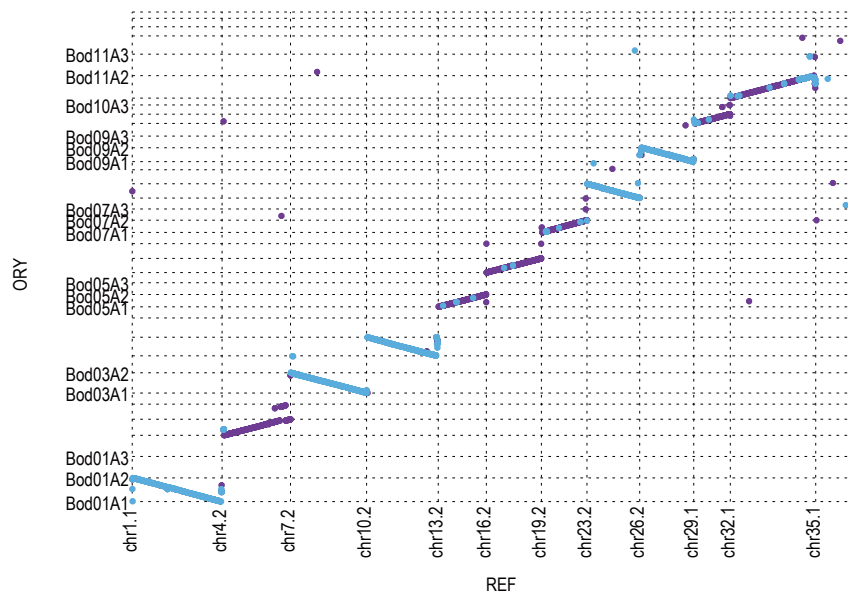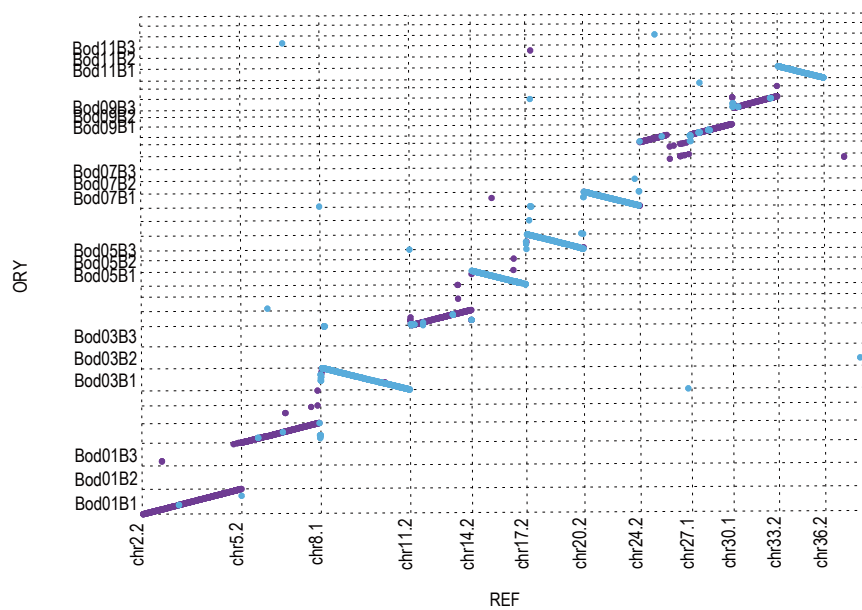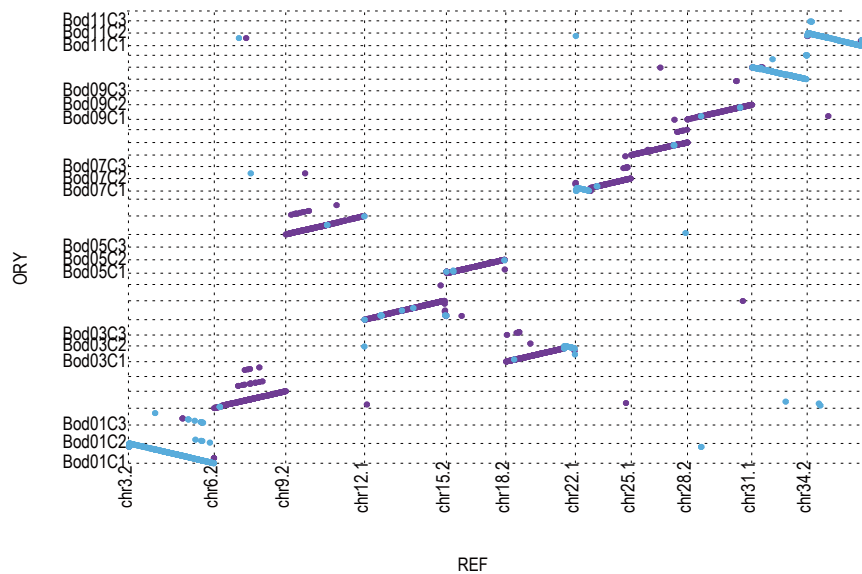

Supplement: Web_Material_uhae250 [file web_material_uhae250.zip › SFigure_4.pdf]

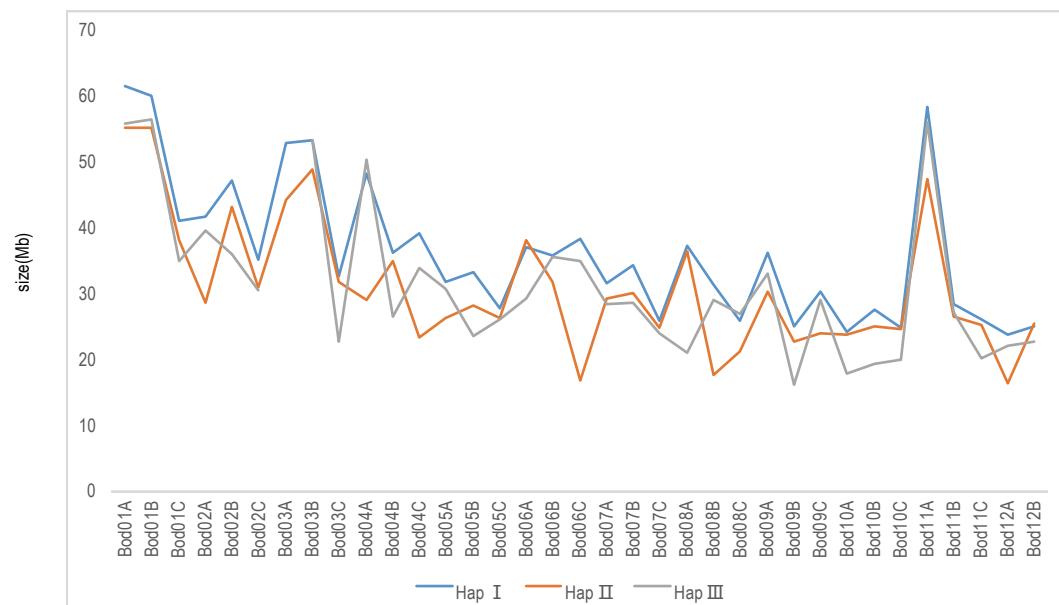

Supplement: Web_Material_uhae250 [file web_material_uhae250.zip › SFigure_5.pdf]

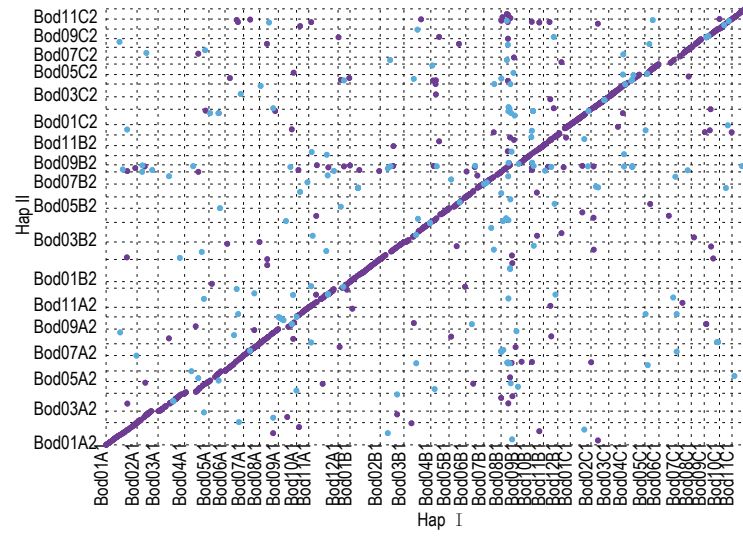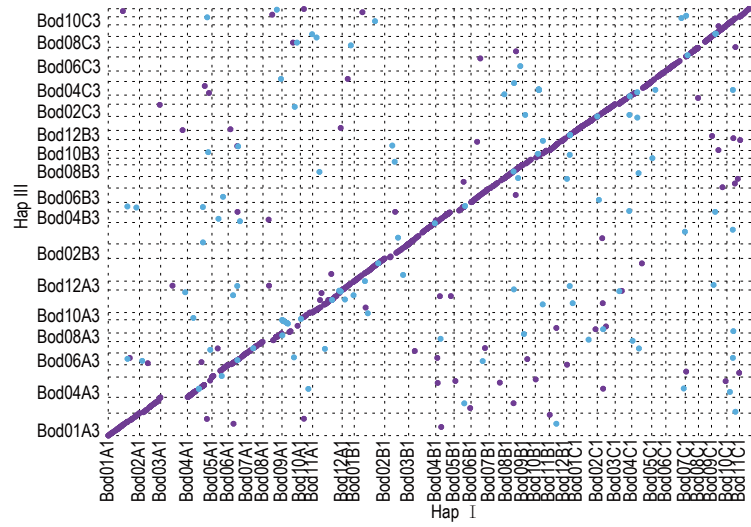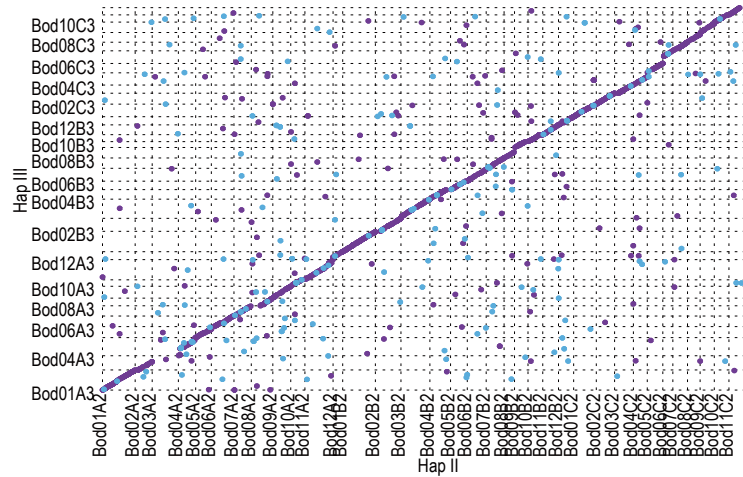

Supplement: Web_Material_uhae250 [file web_material_uhae250.zip › SFigure_6.pdf]

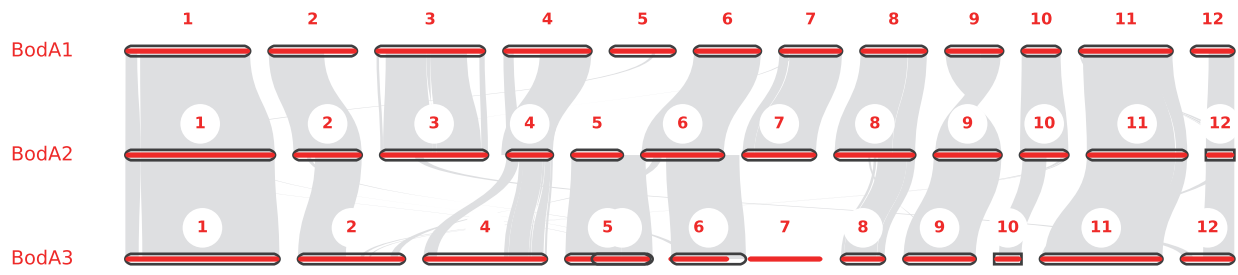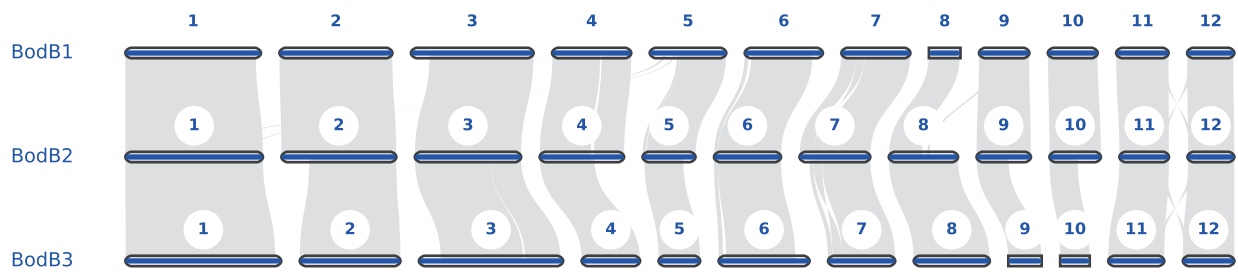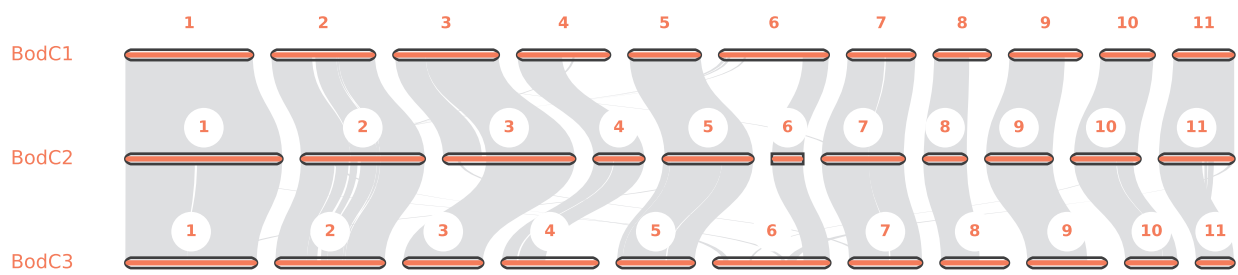

Supplement: Web_Material_uhae250 [file web_material_uhae250.zip › SFigure_7.pdf]

**A**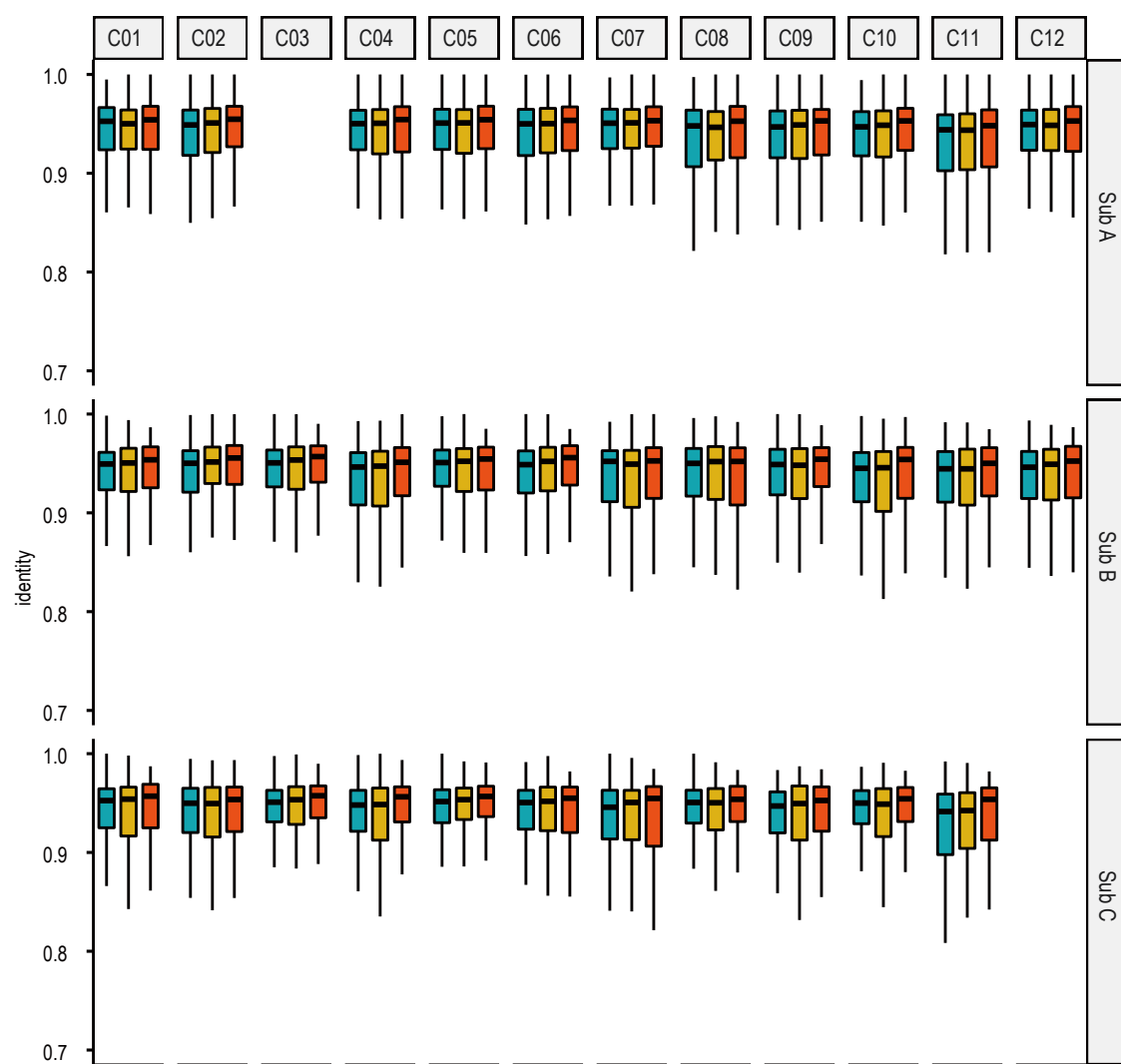**B**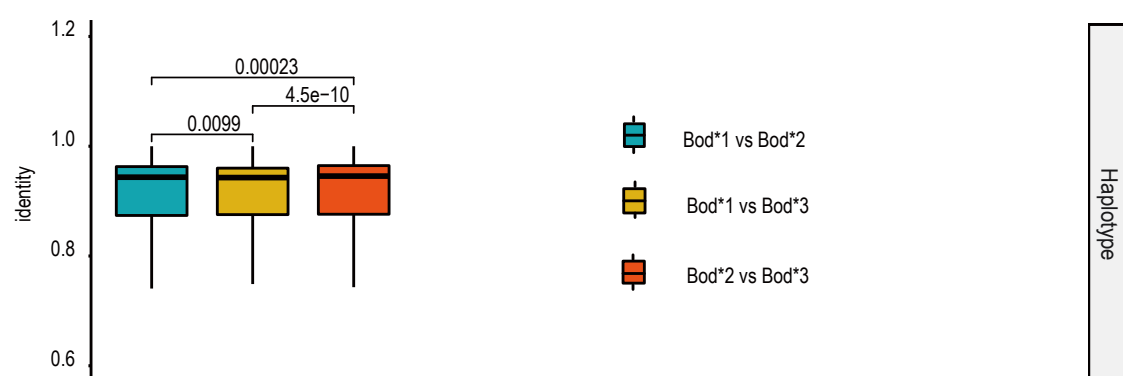

Supplement: Web_Material_uhae250 [file web_material_uhae250.zip › SFigure_8.pdf]

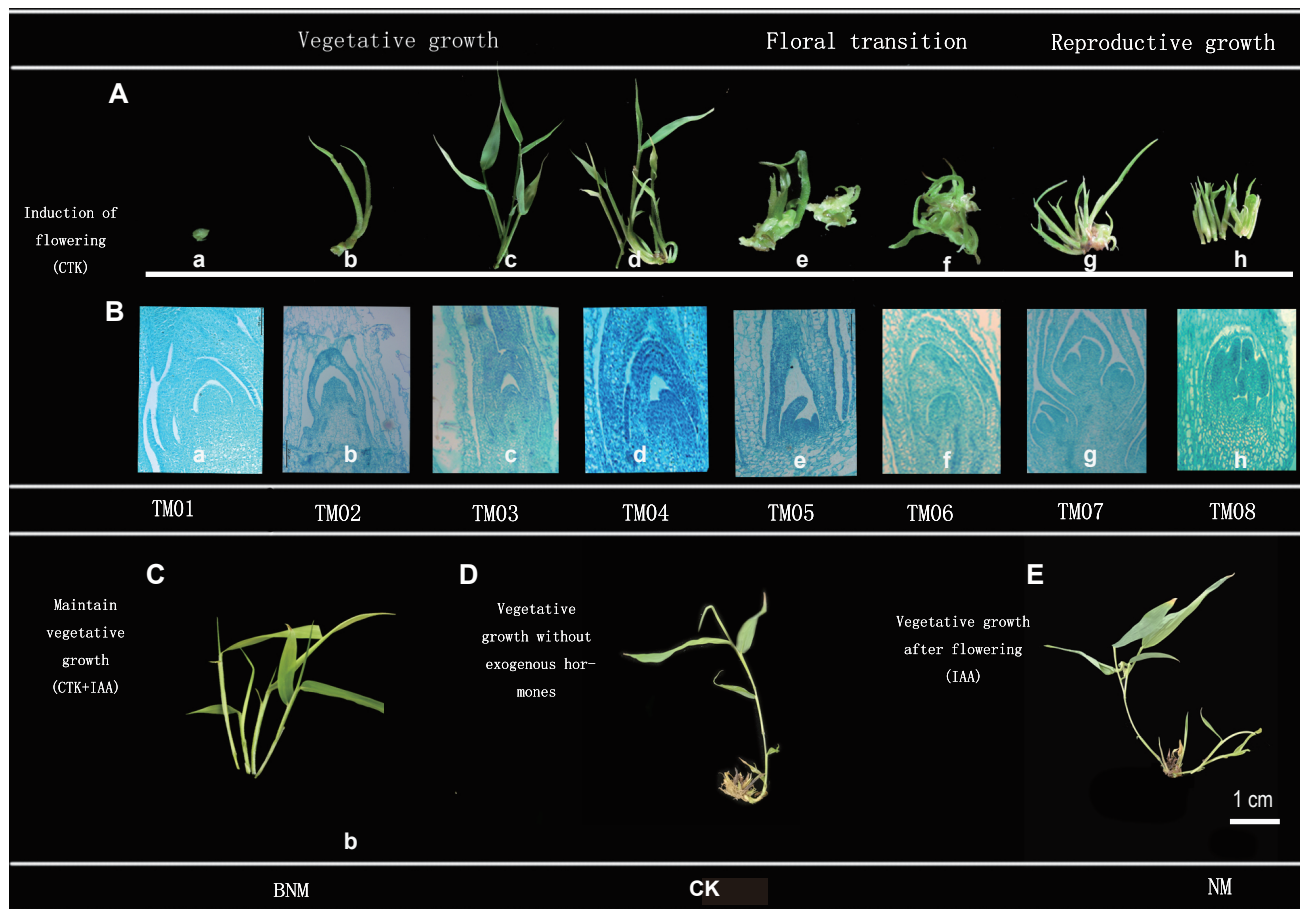

**F**

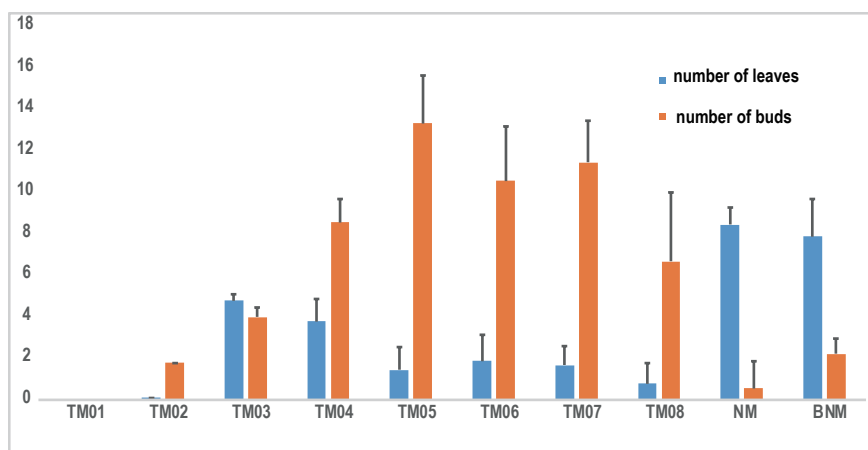

Supplement: Web_Material_uhae250 [file web_material_uhae250.zip › SFigure_9.pdf]

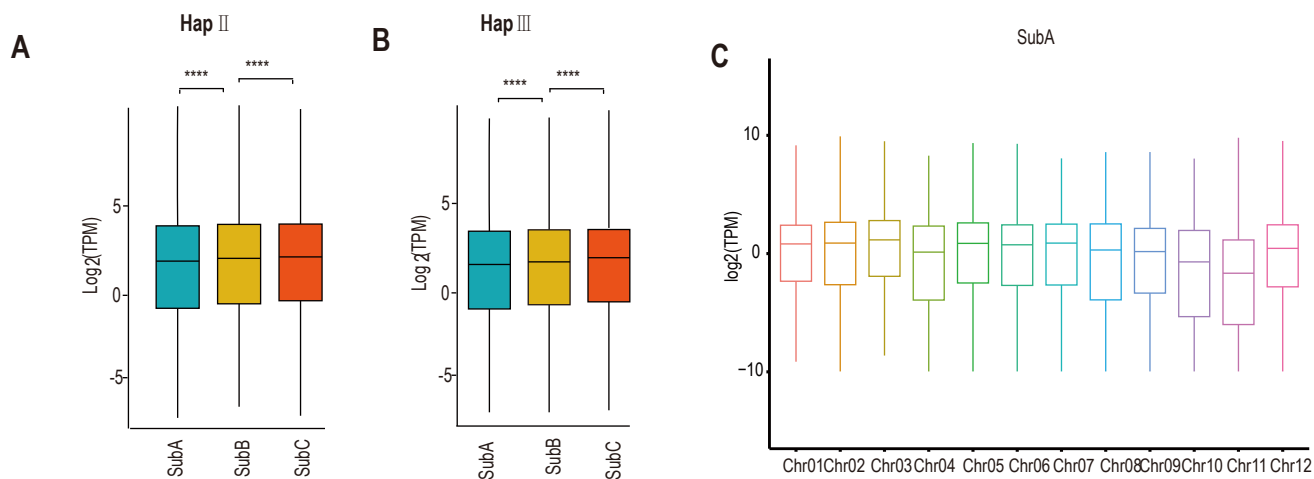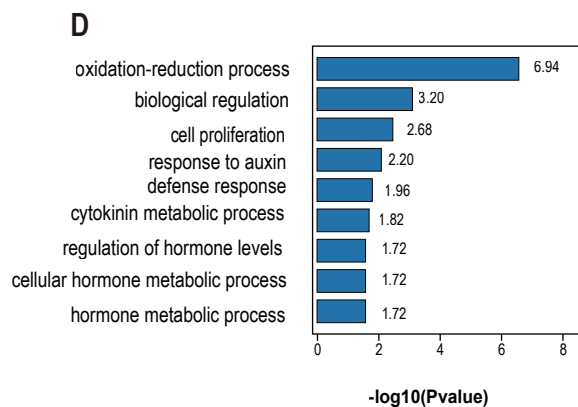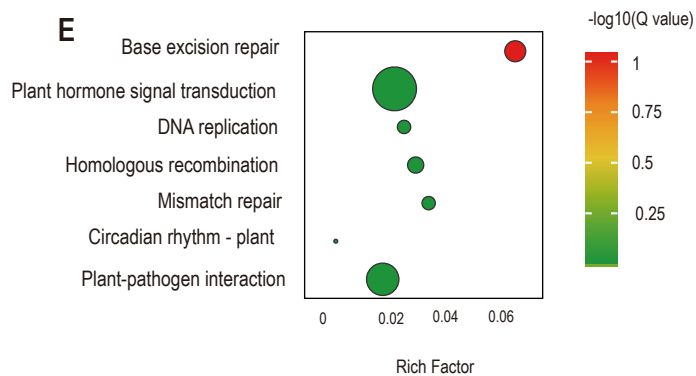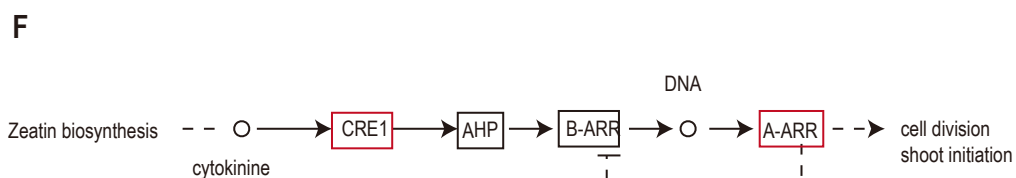

Supplement: Web_Material_uhae250 [file web_material_uhae250.zip › SFigure_10.pdf]

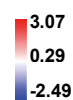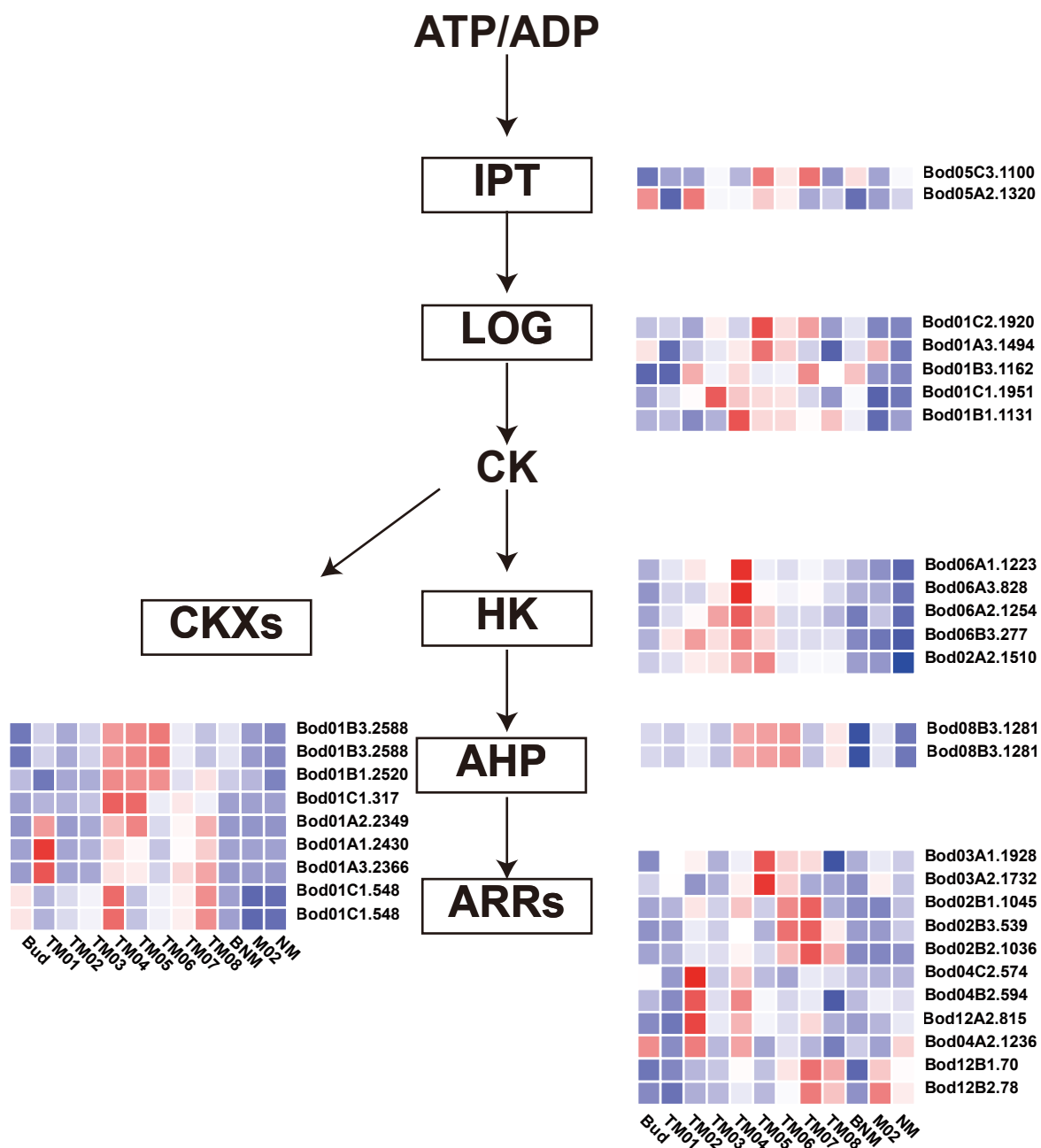

Supplement: Web_Material_uhae250 [file web_material_uhae250.zip › SFigure_11.pdf]

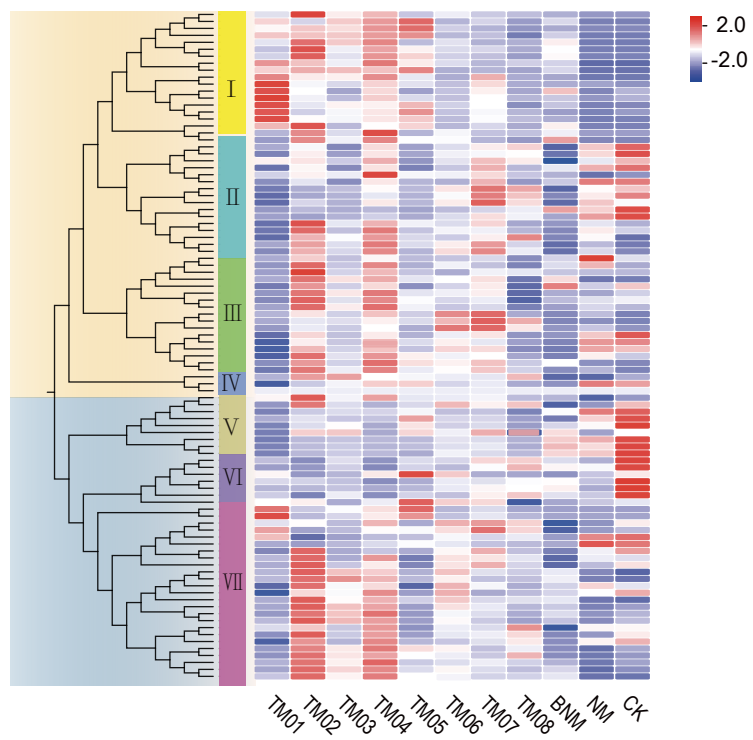

Supplement: Web_Material_uhae250 [file web_material_uhae250.zip › SFigure_12.pdf]

**A**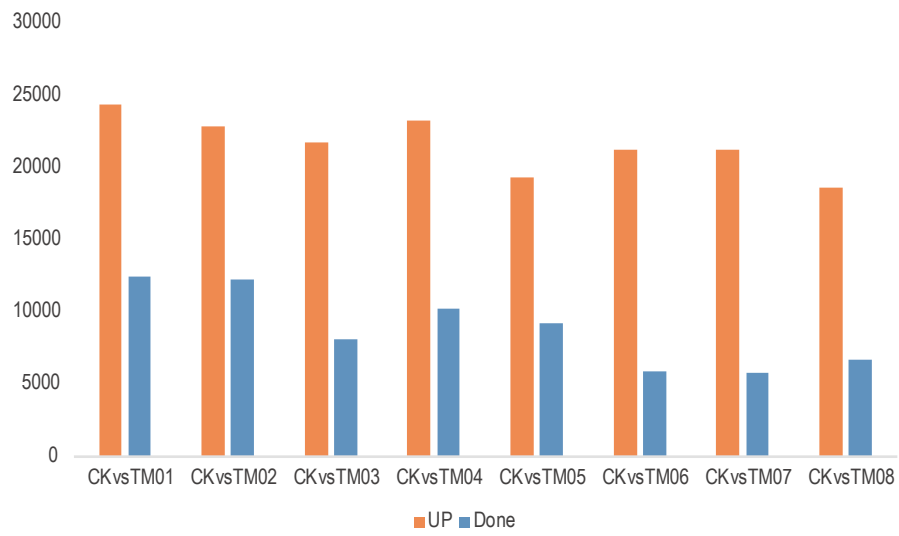**B**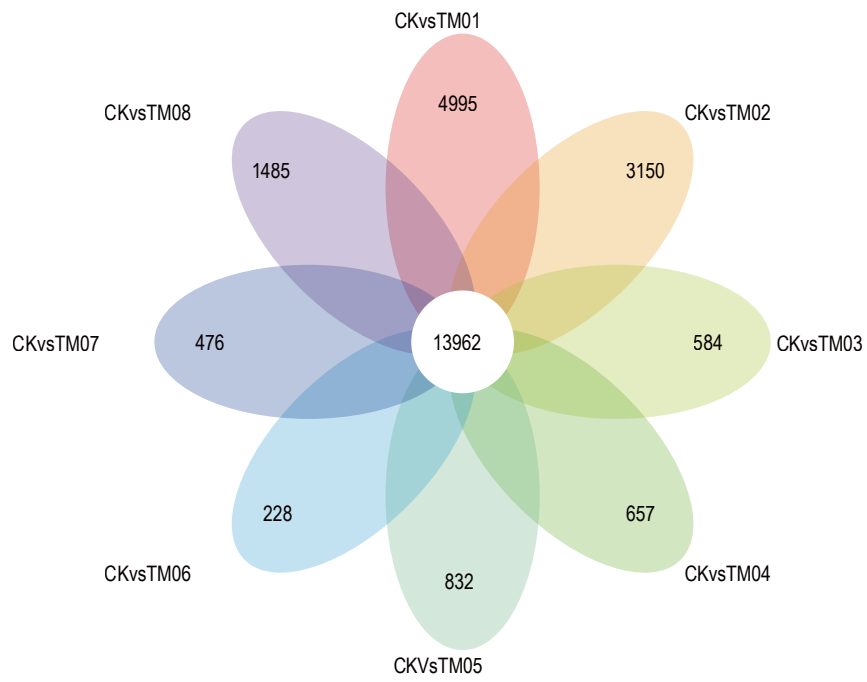

Supplement: Web_Material_uhae250 [file web_material_uhae250.zip › SFigure_13.pdf]

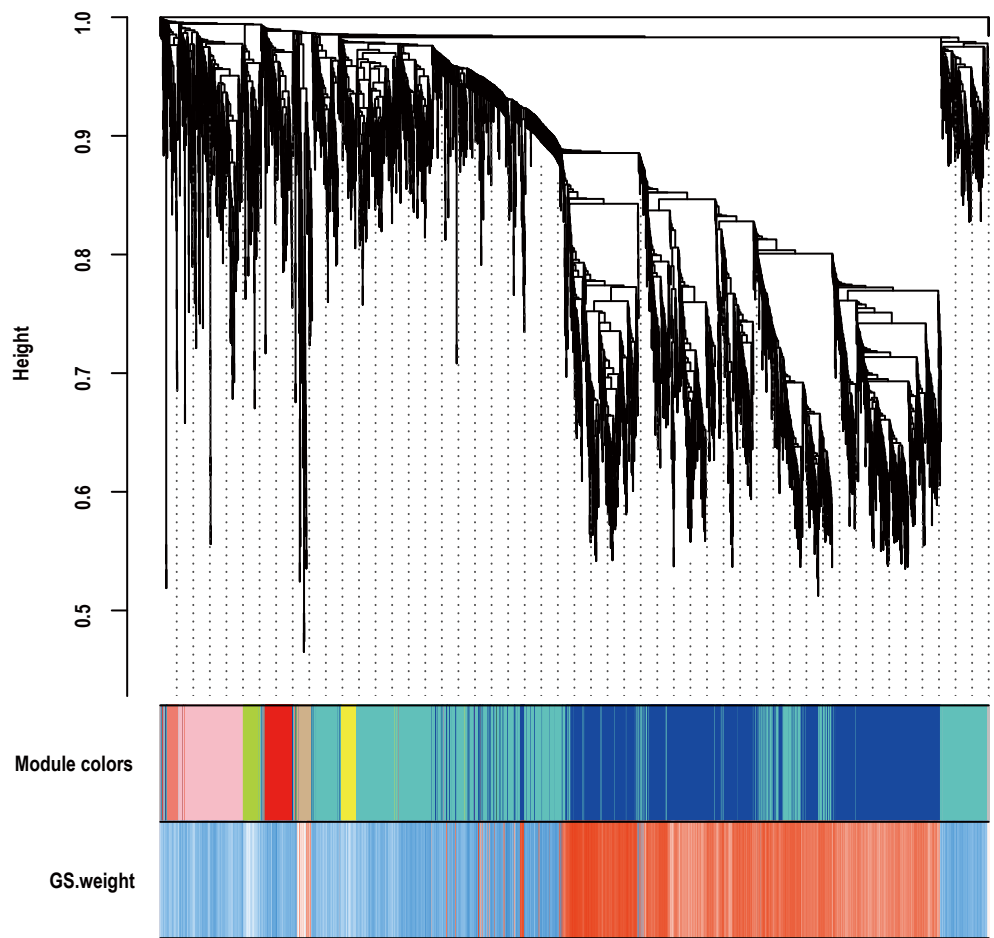

Supplement: Web_Material_uhae250 [file web_material_uhae250.zip › SFigure_14.pdf]

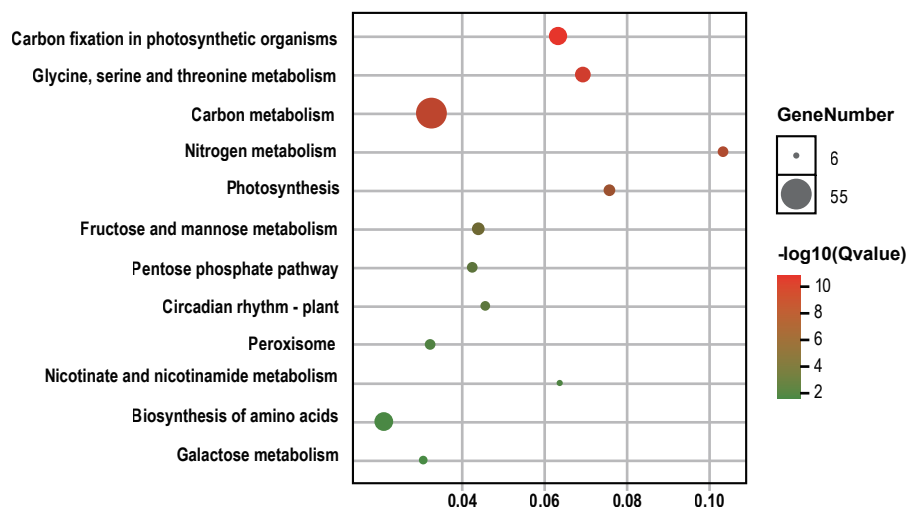

Supplement: Web_Material_uhae250 [file web_material_uhae250.zip › SFigure_15.pdf]

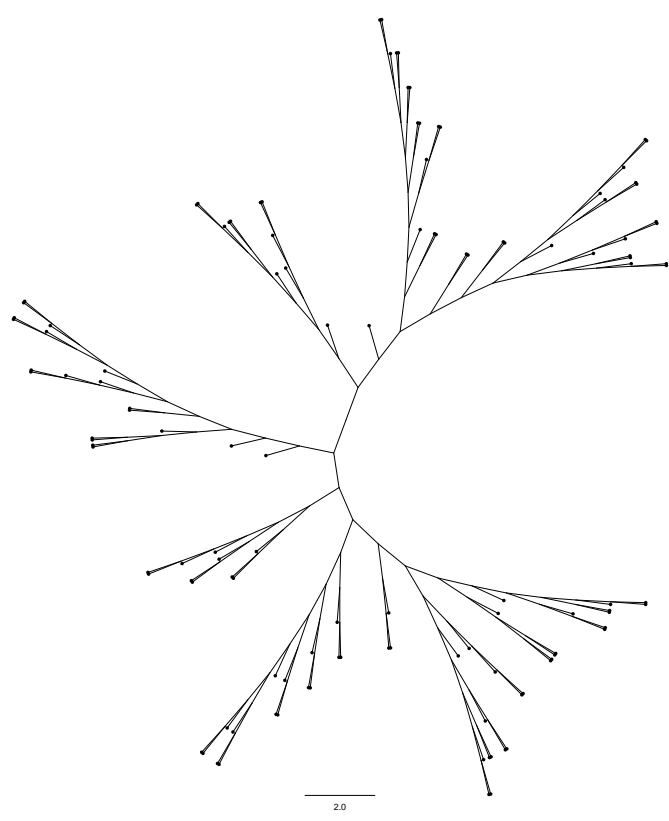

Supplement: Web_Material_uhae250 [file web_material_uhae250.zip › SFigure_16.pdf]

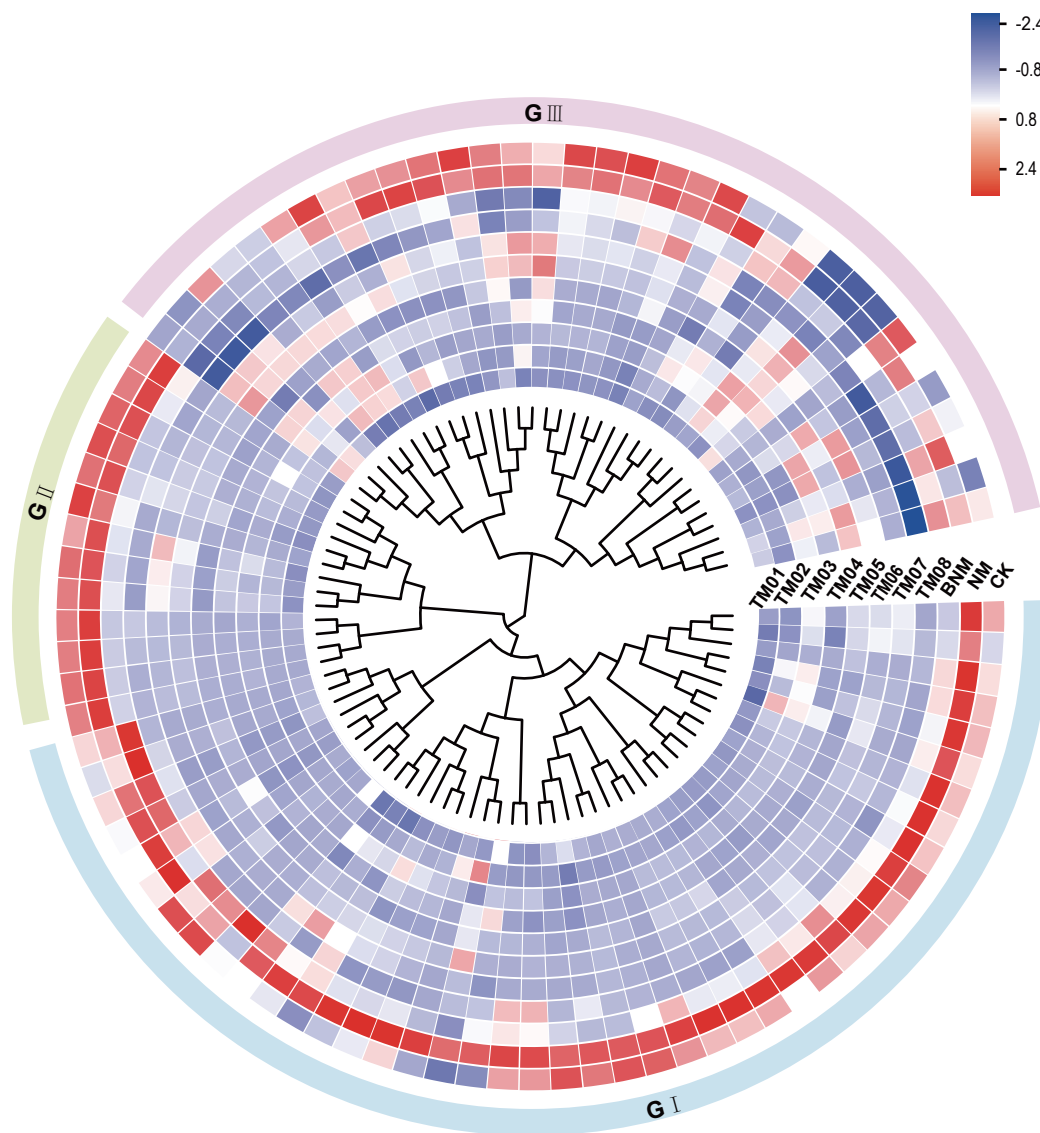

Supplement: Web_Material_uhae250 [file web_material_uhae250.zip › SFigure_17.pdf]

**A**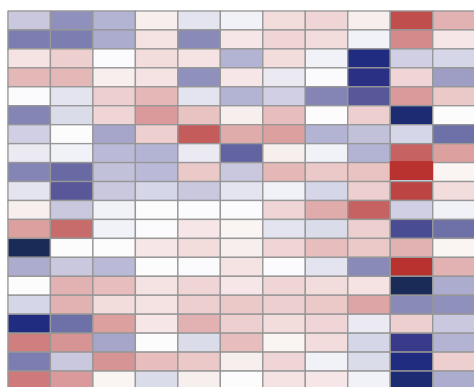**B**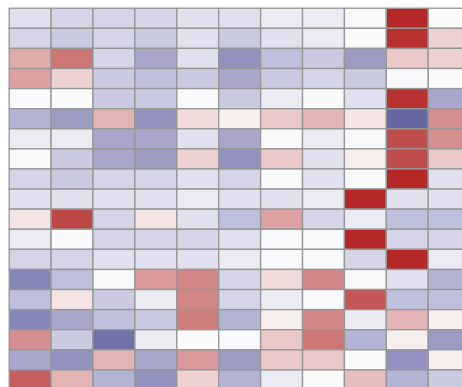**C**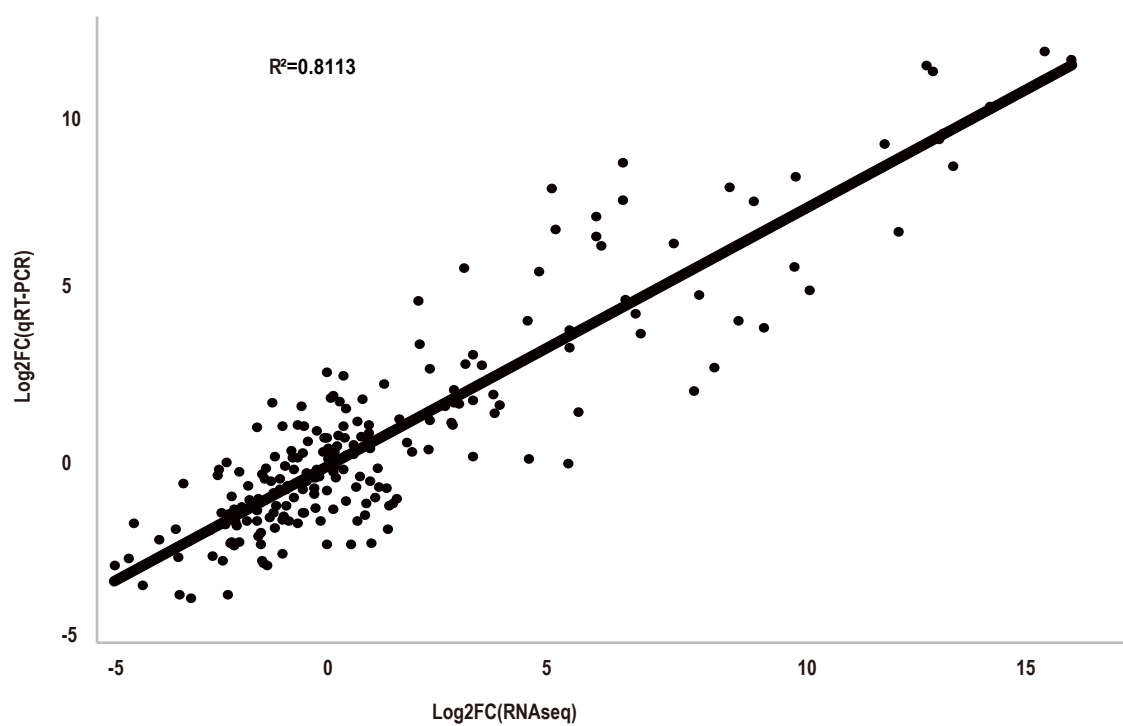

Supplement: Web_Material_uhae250 [file web_material_uhae250.zip › SFigure_18.pdf]

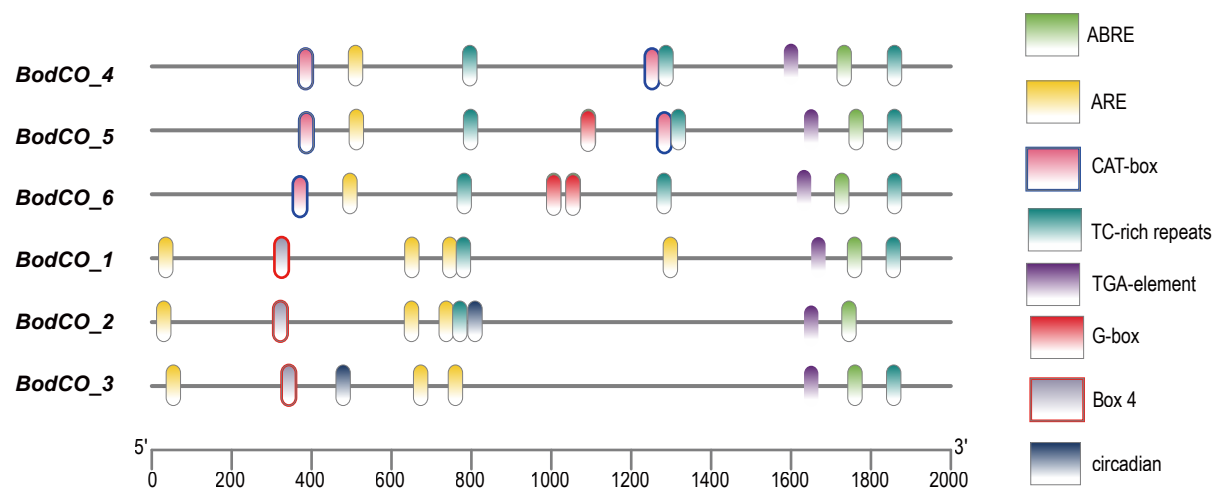

Supplement: Web_Material_uhae250 [file web_material_uhae250.zip › SFigure_19.pdf]
